# Assessing and projecting the global burden of thyroid cancer, 1990–2030: Analysis of the Global Burden of Disease Study

**DOI:** 10.7189/jogh.14.04090

**Published:** 2024-04-05

**Authors:** Qizheng Zhao, Manting Chen, Leiwen Fu, Yan Yang, Yiqiang Zhan

**Affiliations:** 1 Department of Epidemiology, School of Public Health (Shenzhen), Sun Yat-Sen University, Shenzhen, China; 2 Department of Nutrition, School of Public Health (Shenzhen), Sun Yat-Sen University, Shenzhen, China

## Abstract

**Background:**

This study aims to assess the global incidence, mortality, and disability-adjusted life years (DALYs) of thyroid cancer between 1990 and 2030.

**Methods:**

Our study analysed Global Burden of Disease (GBD) 2019 data from 204 countries, spanning 1990–2019. It focused on age-standardised thyroid cancer incidence, mortality, and disability-adjusted life years (DALYs), using the sociodemographic index (SDI) for assessing socioeconomic levels. Generalised additive models (GAMs) projected thyroid cancer trends for 2020–2030.

**Results:**

The global burden of thyroid cancer is predicted to increase significantly from 1990 to 2030. The number of thyroid cancer incidence cases is projected to rise from 233 846.64 in 2019 to 305 078.08 by 2030, representing an approximate 30.46% increase. The ASIR (age-standardised incidence rate) is expected to continue its upward trend (estimated annual percentage change (EAPC) = 0.83). The age-standardised death rate (ASDR) for thyroid cancer is projected to decline in both genders, more notably in women (EAPC = −0.34) compared to men (EAPC = −0.17). The burden of disease escalates with advancing age, with significant regional disparities. Regions with lower SDI, particularly in South Asia, are anticipated to witness substantial increases in thyroid cancer incidence from 2020 to 2030. The overall disease burden is expected to rise, especially in countries with low to middle SDI, reflecting broader socio-economic and health care shifts.

**Conclusions:**

This study highlights significant regional and gender-specific variations in thyroid cancer, with notable increases in incidence rates, particularly in areas like South Asia. These trends suggest improvements in diagnostic capabilities and the influence of socio-economic factors. Additionally, the observed decline in mortality rates across various regions reflects advancements in thyroid cancer management. The findings underline the critical importance of regionally tailored prevention strategies, robust cancer registries, and public health initiatives to address the evolving landscape of thyroid cancer and mitigate health disparities globally.

Thyroid cancer (TC), one of the endocrine malignancies [1], has emerged as a significant public health concern, ranking ninth in cancer incidence globally as of 2020 [2,3]. As of 2019, TC had affected approximately 233.85 thousand individuals globally, resulting in 45.58 thousand deaths and accounting for 1231.84 thousand disability-adjusted life years (DALYs) [4]. From 1990 to 2019, a significant increase in the age-standardised incidence rates of TC has been observed across both sexes, while the age-standardised death and DALY rates have shown a decreasing trend [4].

Primary health care strategies developed by the World Health Organization (WHO), addressing key behavioural and dietary risk factors like hypertension, smoking, and poor nutrition, aim to reduce the impact of noncommunicable diseases, including various cancers [5]. This aligns with the United Nations' Sustainable Development Goals, specifically the target to cut early mortality from these diseases by one-third by 2030, while enhancing mental health and overall well-being [6]. In this context, accurately projecting the future trends of TC is imperative for global health initiatives. Global efforts to lower the mortality, incidence, and DALYs associated with TC are fundamental but the outcomes are yet to be clearly determined. Reliable worldwide predictions are indispensable to effectively quantify TC's burden and long-term effects, and are key to refining prevention strategies, optimising health care services, and shaping informed health policies.

Our study takes a step further from previous research on TC forecasting, which primarily focused on individual countries [7] or have globally extrapolated incidence estimates based solely on the economic stratifications of countries and territories [8]. Generalized Additive Models (GAMs) are adept at navigating the intricacies of nonlinear interdependencies among demographic factors such as age, gender, and geographical region, particularly when dealing with data that deviates from normal distribution [9]. Through this, we are able to blend the clarity of linear models with the necessary flexibility to accurately model the complex patterns of thyroid cancer, enhancing the precision of our forecasts and deepening our understanding of the disease's trajectory. Therefore, we aimed to project both the numbers and age-standardised rates of TC, deaths, and DALYs from 2020 to 2030 in a comprehensive study based on GAMs and GBD data from 1990 to 2019.

## METHODS

### Data source

The GBD 2019 Study is a global epidemiology programme designed to assess the burden of disease, injury, and risk factors globally, regionally, and nationally. The GBD Study offers detailed data on incidence, prevalence, mortality, and DALYs for over 350 diseases across 204 countries and territories [10].

TC was ascertained by the International Classification of Diseases Ninth Revision (ICD-9: 193-193.9 and 226-226.9) and ICD-10 (C73-C73.9, D09.3, D09.8, D34-D34.9, and D44.0) [11–14].

Annual incidence and mortality data were extracted for thyroid cancer and population size by sex, age groups, income groups, and countries and territories. These data were obtained from the Global Health Data Exchange (GHDE). All estimates included 95% uncertainty intervals (UIs).

The sociodemographic index (SDI), which is a composite indicator that measures the level of social and economic development of a region based on per capita income, average years of schooling, and fertility rate, was used as a sociodemographic indicator for countries. The sociodemographic index is stratified into five categories, which represent different levels of socioeconomic development: low SDI (0 to 0.45), low-middle SDI (>0.45 to 0.55), middle SDI (>0.55 to 0.70), high-middle SDI (>0.70 to 0.85), and high SDI (>0.85 to 1).

### Statistical analysis

Our study utilised historical population data from 1990 to 2019 for each country to estimate population changes, employing a logarithmic model:

*ln(pnum) = a × year + b*

where pnum represents the population size, a is the coefficient of the year, b denotes the intercept, and year refers to the calendar year.

GAMs were used to predict the numbers and rates of TC cases, deaths, and DALYs. The GAM was specified as:

*ln(number) = s[ln(pnum)] + s(c) + s(year) + s(e) + r*

where number refers to the count of TC cases, deaths, or DALYs, pnum is the population; c denotes the median age in each age group, year represents the calendar year, and e is the difference between the calendar year and the mid-value of the age group with r being the intercept. The s denotes a smoothing spline function.

To enhance the accuracy and reliability of our projections for trends through 2030 for TC, we utilised the bootstrap method to generate predicted values and their 95% confidence intervals (CIs). We analysed temporal trends in TC by calculating age-standardised rates and their Estimated Annual Percentage Changes (EAPCs). Trends were classified as increasing or decreasing based on the direction of the EAPC values and their 95% CIs.

All analyses were conducted using R statistical software (version 4.0), with a *P* < 0.05 set as the threshold for statistical significance. Our study followed the Cross-Sectional Study Health Estimates Guidelines for Accurate and Transparent Reporting (GATHER) to ensure the accuracy and transparency of the study.

## RESULTS

### Prediction of the Global Trend of Burden of thyroid cancer, 2020–2030

**Figure 1:**
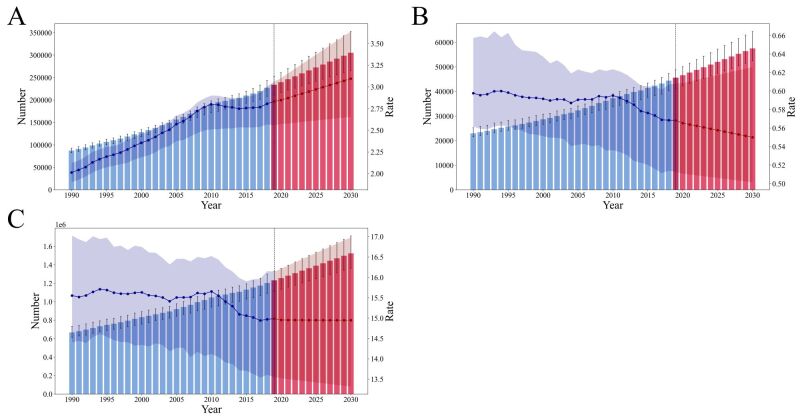
Trends in global thyroid cancer burden from 1990 to 2019 and Projected Rates from 2020 to 2030. **Panel A.** Age-standardised incidence rate. **Panel B.** Age-standardised death rate. **Panel C.** Disability-adjusted life-year.

The ASIR is anticipated to rise annually from 2020 to 2030 (EAPC = 0.83, 95% CI = 0.32, 1.61), while the ASDR is expected to decrease (EAPC = −0.28, 95% CI = −0.18, 0.28), and the trend in the age-standardised DALY rate is predicted to be relatively stable (EAPC = 0.00, 95% CI = −0.15, 0.46) (**Table 1**, Figures S1–S2 in the **Online Supplementary Document**). The number of observed cases of incidence, deaths, and DALYs of thyroid cancer has shown an upward trend from 1990 to 2019 (**Figure 1**, Table S1 in the **Online Supplementary Document**). This trend is projected to continue from 2020 to 2030. By 2030, the number of thyroid cancer incidence cases is projected to increase from 233 846.64 in 2019 (95% CI = 211 636.89, 252 806.55) to 305 078.08 (95% CI = 265 839.75, 352 293.15) (Table S1 in the **Online Supplementary Document**). The ASIR is expected to increase to 2.85 / 100 000 in 2020 (95% CI = 2.57 / 100 000, 3.10 / 100 000) and rise further to 3.09 / 100 000 in 2030 (95% CI = 2.65 / 100 000, 3.64 / 100 000) (**Table 1**, **Figure 1**, panel A). The ASDR is predicted to decrease from 0.57 / 100 000 in 2020 (95% CI = 0.51, 0.61) to 0.55 / 100 000 in 2030 (95% CI = 0.50, 0.63) (**Table 1**, **Figure 1**, panel B), and the age-standardised DALY rate in 2030 is expected to be 14.95 (95% CI = 13.32, 16.98) per 100 000 persons, almost consistent with that in 2020 (DALY rate = 14.95 per 100 000 persons, 95% CI = 13.52, 16.21) (**Table 1**, **Figure 1**, panel C).

**Table 1:** Trends in global thyroid cancer burden from 1990 to 2019 and observed and projected rates from 2020 to 2030.

	1990–2019 EAPC (95%CI)			2020–2030 EAPC (95%CI)		
Location	ASIR	ASDR	Age-standardised DALY rate	ASIR	ASDR	Age-standardised DALY rate
Global	0.41 (0.28, 0.53)	−0.05 (−0.16, 0.02)	−0.04 (−0.15, 0.05)	0.83 (0.32, 1.61)	−0.28 (−0.18, 0.28)	−0.00 (−0.15, 0.46)
Sex	–	–	–	–	–	–
*Female*	0.32 (0.19, 0.47)	−0.15 (−0.25, −0.06)	−0.13 (−0.25, −0.01)	0.84 (0.41, 1.65)	−0.34 (−0.75, 0.24)	−0.06 (−0.34, 0.50)
*Male*	0.64 (0.44, 0.83)	0.17 (−0.00, 0.32)	0.15 (−0.02, 0.31)	0.87 (0.68, 1.62)	−0.17 (−0.01, 0.64)	0.08 (0.26, 0.55)
SDI	–	–	–	–	–	–
*High SDI*	0.35 (0.23, 0.48)	−0.15 (−0.22, −0.11)	−0.11 (−0.18, −0.06)	−0.37 (−1.67, 1.19)	−0.34 (−0.26, −0.16)	−0.37 (−0.54, −0.36)
*High-middle SDI*	0.34 (0.21, 0.50)	−0.21 (−0.27, −0.14)	−0.20 (−0.26, −0.13)	0.69 (−0.39, 1.68)	−0.79 (−1.47, −0.16)	−0.53 (−1.19, −0.05)
*Middle SDI*	0.86 (0.55, 1.13)	0.06 (−0.14, 0.20)	0.05 (−0.14, 0.18)	1.90 (1.55, 2.47)	−0.62 (−1.02, 0.33)	−0.22 (−0.47, 0.63)
*Low-middle SDI*	0.69 (0.39, 0.97)	0.12 (−0.07, 0.28)	0.10 (−0.09, 0.27)	2.15 (2.05, 2.44)	0.20 (−0.05, 0.61)	0.32 (0.04, 0.71)
*Low SDI*	0.29 (0.02, 0.65)	−0.01 (−0.20, 0.20)	−0.07 (−0.27, 0.17)	1.73 (1.88, 1.98)	0.27 (−0.00, 0.25)	0.14 (0.01, 0.15)
Region	–	–	–	–	–	–
*Andean Latin America*	1.06 (0.50, 1.66)	0.20 (−0.16, 0.52)	0.13 (−0.17, 0.44)	1.07 (0.17, 2.59)	−0.92 (−0.55, 0.68)	−0.62 (−0.87, 0.92)
*Australasia*	0.92 (0.44, 1.52)	0.11 (−0.07, 0.22)	0.18 (−0.01, 0.32)	−0.87 (−7.71, 2.80)	−0.63 (−0.86, −0.36)	−0.72 (−1.16, −0.09)
*Caribbean*	0.43 (0.19, 0.69)	0.06 (−0.09, 0.23)	0.07 (−0.10, 0.26)	0.49 (−1.71, 2.43)	−0.34 (−2.38, 1.05)	−0.22 (−2.10, 1.18)
*Central Asia*	0.18 (0.02, 0.38)	−0.03 (−0.18, 0.10)	−0.13 (−0.25, 0.00)	0.17 (−1.55, 1.56)	−2.20 (−3.96, −0.54)	−1.73 (−3.80, 0.10)
*Central Europe*	0.02 (-0.13, 0.23)	−0.40 (−0.47, −0.26)	−0.40 (−0.48, −0.23)	0.83 (−2.42, 2.40)	−0.32 (−3.01, 1.57)	−0.09 (−3.44, 1.48)
*Central Europe, Eastern Europe, and Central Asia*	0.42 (0.28, 0.60)	−0.13 (−0.20, −0.05)	−0.11 (−0.18, −0.02)	−0.20 (−3.21, 1.70)	−0.96 (−2.36, 0.46)	−0.88 (−2.73, 0.46)
*Central Latin America*	0.61 (0.38, 0.87)	−0.02 (−0.14, 0.12)	−0.01 (−0.14, 0.14)	1.25 (−1.40, 3.37)	0.07 (−1.06, 1.97)	0.17 (−2.08, 2.25)
*Central sub-Saharan Africa*	0.11 (−0.18, 0.45)	−0.04 (−0.25, 0.21)	−0.08 (−0.31, 0.17)	1.33 (0.68, 1.54)	0.03 (0.13, 0.11)	−0.06 (−0.46, −0.08)
*East Asia*	0.96 (0.54, 1.52)	−0.08 (−0.31, 0.13)	−0.11 (−0.32, 0.09)	1.98 (0.93, 2.64)	−1.23 (−2.01, −0.11)	−0.62 (−1.62, −0.20)
*Eastern Europe*	0.86 (0.60, 1.16)	0.09 (−0.03, 0.22)	0.17 (0.03, 0.30)	−0.43 (−5.24, 2.37)	−1.09 (−4.13, 1.04)	−1.04 (−3.66, 0.64)
*Eastern sub-Saharan Africa*	0.13 (−0.19, 0.61)	−0.10 (−0.32, 0.18)	−0.20 (−0.42, 0.12)	1.90 (1.90, 2.12)	0.25 (−0.08, −0.01)	0.13 (0.19, 0.06)
*High-income*	0.28 (0.16, 0.40)	−0.18 (−0.24, −0.14)	−0.15 (−0.20, −0.10)	−0.51 (−1.97, 1.21)	−0.32 (−0.39, −0.18)	−0.43 (−0.59, −0.20)
*High-income Asia Pacific*	0.50 (0.13, 0.77)	−0.16 (−0.34, −0.08)	−0.11 (−0.34, −0.02)	−1.71 (−1.40, 0.40)	−1.13 (−1.03, −0.85)	−1.38 (−0.78, −1.08)
*High-income North America*	0.36 (0.17, 0.57)	0.10 (0.06, 0.14)	0.11 (0.05, 0.16)	−0.72 (−6.38, 2.62)	−0.14 (−0.01, −0.07)	−0.45 (−0.75, −0.31)
*Latin America and Caribbean*	0.49 (0.33, 0.67)	−0.06 (−0.14, 0.04)	−0.06 (−0.15, 0.04)	0.70 (−1.00, 2.29)	−0.57 (−1.33, 0.84)	−0.46 (−1.54, 1.03)
*North Africa and Middle East*	1.03 (0.67, 1.55)	0.01 (−0.16, 0.23)	0.03 (−0.14, 0.28)	2.14 (1.69, 2.64)	−0.36 (−1.23, 0.02)	−0.02 (−0.63, 0.46)
*Oceania*	0.24 (−0.03, 0.59)	0.06 (−0.16, 0.30)	0.05 (−0.16, 0.31)	1.02 (0.26, 1.47)	0.19 (−0.39, 0.28)	0.07 (−0.59, 0.47)
*South Asia*	0.81 (0.35, 1.20)	0.14 (−0.10, 0.36)	0.15 (−0.09, 0.36)	2.49 (1.84, 3.43)	0.56 (−0.15, 1.29)	0.60 (0.18, 1.66)
*Southeast Asia*	0.65 (0.39, 0.97)	0.04 (−0.12, 0.21)	0.01 (−0.14, 0.18)	1.63 (0.90, 2.22)	−0.36 (−0.54, 0.57)	−0.27 (−0.28, 0.51)
*Southeast Asia, East Asia, and Oceania*	0.86 (0.54, 1.23)	−0.04 (−0.23, 0.12)	−0.05 (−0.23, 0.09)	1.90 (1.08, 2.41)	−0.86 (−1.16, 0.11)	−0.38 (−0.38, 0.28)
*Southern Latin America*	0.26 (−0.02, 0.65)	−0.23 (−0.29, −0.13)	−0.23 (−0.30, −0.12)	0.98 (−6.71, 4.25)	−0.17 (−0.50, 0.07)	−0.05 (−0.53, 0.42)
*Southern sub-Saharan Africa*	0.13 (−0.02, 0.31)	0.06 (−0.07, 0.19)	0.01 (−0.11, 0.16)	−1.15 (−1.56, −0.22)	−3.61 (−2.97, −2.81)	−3.07 (−2.81, −2.31)
*Sub-Saharan Africa*	0.13 (−0.13, 0.50)	−0.07 (−0.25, 0.14)	−0.15 (−0.34, 0.11)	1.41 (1.37, 1.94)	−0.20 (−0.49, −0.13)	−0.23 (−0.69, 0.09)
*Tropical Latin America*	0.19 (0.12, 0.30)	−0.24 (−0.28, −0.18)	−0.23 (−0.28, −0.16)	−0.47 (−0.94, −0.03)	−1.57 (−2.09, −1.00)	−1.59 (−1.94, −1.01)
*Western Europe*	0.08 (−0.06, 0.25)	−0.33 (−0.37, −0.28)	−0.29 (−0.34, −0.24)	0.25 (−2.93, 2.25)	−0.19 (−0.36, 0.06)	−0.08 (−0.11, 0.35)
*Western sub-Saharan Africa*	0.20 (0.00, 0.46)	−0.03 (−0.17, 0.15)	−0.04 (−0.20, 0.15)	0.67 (0.36, 0.90)	−0.63 (−2.24, −0.40)	−0.70 (−1.36, −0.39)

ASDR – age-standardised death rate, ASIR – age-standardised incidence rate, CI – confidence interval, DALY – disability-adjusted life years, EAPC – estimated annual percentage change, SDI – sociodemographic index

### Prediction of the global trend of burden of thyroid cancer by gender, 2020–2030

**Figure 2:**
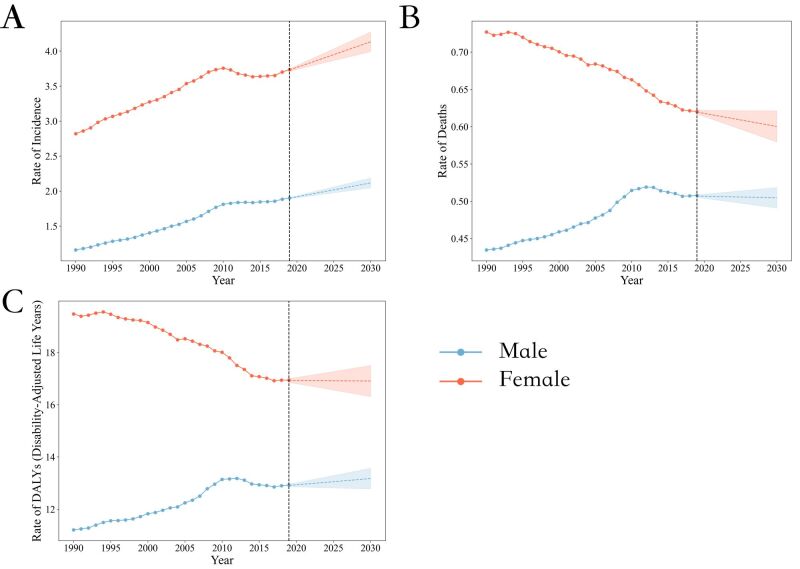
Trends in global thyroid cancer burden from 1990 to 2030, by genders. **Panel A.** Age-standardised incidence rate. **Panel B.** Age-standardised death rate. **Panel C.** Disability-adjusted life-year.

Projected trends indicate an increase in thyroid cancer incidence alongside improved survival outcomes for both genders from 2020 to 2030. It is predicted that by 2030, the ASIR for women will rise from 3.76 / 100 000 in 2020 (95% CI = 3.34 / 100 000, 4.16 / 100 000) to 4.08 / 100 000 (95% CI = 3.48 / 100 000, 4.90 / 100 000), and for men, it will increase from 1.91 / 100 000 (95% CI = 1.73 / 100 000, 2.10 / 100 000) to 2.09 / 100 000 (95% CI = 1.85 / 100 000, 2.47 / 100 000). From 1990 to 2019, there was an upward trend in ASIR for both men and women, and this upward trend is expected to continue from 2020 to 2030. The EAPC for ASIR is 0.84 (95% CI = 0.41, 1.65) for men, and 0.87 (95% CI = 0.68, 1.62) for women (**Table 1**, **Figure 2**, panel A).

In contrast, a declining trend for ASDR is predicted for both men and women from 2020 to 2030. The EAPC for ASDR in women is −0.34 (95% CI = −0.75, 0.24), which is doubling the rate of decline compared to men, whose EAPC for ASDR is −0.17 (95% CI = −0.01, 0.64) (**Table 1**, **Figure 2**, panel B).

The DALY rate for women is predicted to decrease slightly from 16.90 (95% CI = 14.66, 18.72) per 100 000 persons in 2020 to 16.81 (95% CI = 14.17, 19.68) per 100 000 persons in 2030, with an EAPC of −0.06 (95% CI = −0.34, 0.50). For men, the age-standardised DALY rate is expected to increase marginally from 12.90 (95% CI = 11.74, 14.08) per 100 000 persons in 2020 to 13.00 (95% CI = 12.05, 14.88) per 100 000 persons in 2030, with an EAPC of 0.08 (95% CI = 0.26, 0.55), indicating a slight upward trend from 2020 to 2030 (**Table 1**, **Figure 2**, panel C).

### Prediction of the global trend of burden of thyroid cancer by age, 2020–2030

**Figure 3:**
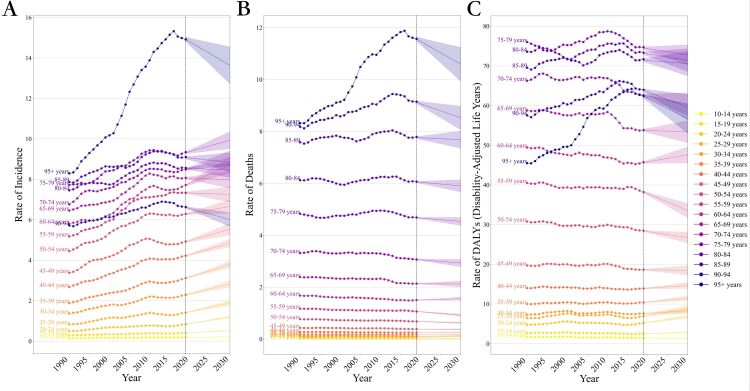
Trends in global thyroid cancer burden from 1990 to 2030, by ages. **Panel A.** Incidence rate. **Panel B.** Death rate. **Panel C.** Disability-adjusted life-year.

The global trend of thyroid cancer from 2020 to 2030 forecasts a nuanced shift, with incidence rates increasing in the younger to middle-aged populations while predicting a complex pattern of changes in death and DALY rates across different age groups. The forecast for the period 2020–2030 indicates significant variations in the age distribution of the thyroid cancer. From 1990 to 2030, a general upward trend in ASRs over ages has been observed globally (**Figure 3**).

The global incidence rate of thyroid cancer generally increased with age, particularly noticeable in the age subgroups of 10–79 years. However, the incidence rates for the 80–84 and 85–89 age subgroups were lower than expected, similar to the levels of the 75–79 age group. The incidence rate for the 90–94 age group was even lower, comparable to the 55–59 age group. The forecast for 2020–2030 suggests an upward trend for the four age groups over 80, while a declining trend is expected for other age groups. Notably, the 95+ age group has shown a rapid increase since 1990, reaching its peak in 2016 with an incidence rate of 15.32 / 100 000 (95% CI = 10.89 / 100 000, 17.80 / 100 000) (Table S2 in the **Online Supplementary Document**). However, a rapid decline is predicted post-2016, with the rate estimated to be 13.83 / 100 000 (95% CI = 8.88 / 100 000, 17.12 / 100 000) by 2030, still higher than the burden in other age groups during the entire period (**Figure 3**, panel A, Table S2 in the **Online Supplementary Document**).

From 1990 to 2019, the global death rate of thyroid cancer consistently increased a trend expected to continue from 2020 to 2030. The fluctuations in the death rate for those under 89 years were relatively small throughout 1990–2030. For the 90–95 and 95+ age groups, the death rate is predicted to initially increase and then decrease. The death rate for the 90–95 age group reached its maximum of 9.45 (95% CI = 7.40, 10.61) in 2013 and is expected to decline to 8.38 (95% CI = 5.99, 9.68) by 2030. The 95+ age group's death rate peaked at 11.88 (95% CI = 8.46, 13.68) in 2016, with a predicted decrease to 10.64 (95% CI = 7.52, 12.65) by 2030 (**Figure 3**, panel B, Table S3 in the **Online Supplementary Document**).

The global DALY rate for thyroid cancer is predicted to gradually increase across the 14 age groups between 10–79 years from 1990 to 2030. However, for the four age groups over 80 years, the DALY rate is expected to decrease over time. For the 2020–2030 period, a downward trend in the DALY rate is forecasted for the age groups over 80, as well as the 50–54 and 55–59 age groups. In contrast, an upward trend is predicted for other age groups during this period (**Figure 3**, panel C, Table S4 in the **Online Supplementary Document**).

**Figure 4:**
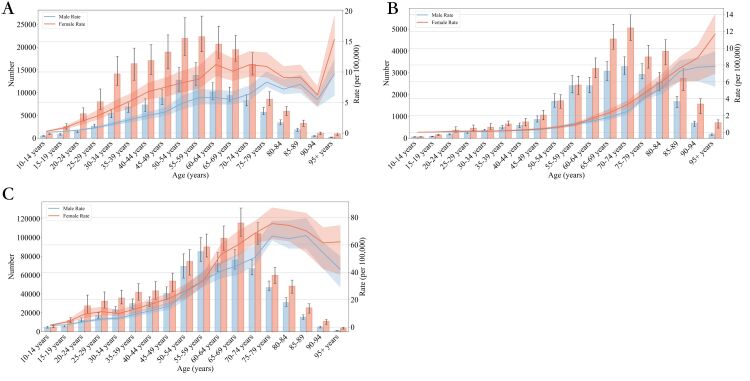
The numbers and rate of global thyroid cancer cases, deaths, and disability-adjusted life years between 1990 and 2030, by ages. **Panel A.** Incidence rate. **Panel B.** Death rate. **Panel C.** Disability-adjusted life-year.

In predicting the distribution of disease case numbers and burden rates for 2030 across different age groups, we find that the three indicators generally show higher numbers in middle age groups and smaller numbers at the extremes. Overall, the disease burden for females is greater than that for males across all age groups. Specifically, the highest number of incidence cases is predicted for women aged 55–59, with an expected incidence number of 22 300.03 (95% CI = 18 982.89, 26 841.07). For death numbers, the highest is projected in women aged 70–74, estimated at 5054.30 (95% CI = 4476.16, 5668.03). The highest predicted number for DALY is in women aged 65–69, at 115 435.47 (95% CI = 101 150.68, 131 379.19) (**Figure 4**).

It is noteworthy that in terms of rate distribution predictions for 2030 across different age groups, the death rate increases with advancing age groups (with the highest being 11.65 for 95+ years). However, the incidence and DALY rates follow specific patterns. For the incidence rate, there is an increasing trend from ages 10–64, followed by a decline until 94 years, and then a significant increase again in the 95+ age group (with the highest being 15.34, for 95+ years). For the DALY rate, there is an increasing trend from ages 10–79, followed by a continuous decline, with the female group in the 95+ years category reaching the highest DALY rate at 75.55 (95% CI = 64.26, 87.09) (**Figure 4****,** Tables S2–S4 and Figures S3–S5 in the **Online Supplementary Document**).

### Prediction of global thyroid cancer burden distribution among different regions and countries for 2020–2030

The forecast for 2020 to 2030 highlights a pronounced increase in the thyroid cancer burden in South Asia, with this region facing the highest growth in incidence, death rates, and DALYs, contrasting with mixed trends across other global regions and countries. It is predicted that South Asia will experience the most significant increase in thyroid cancer burden from 2020 to 2030. The EAPC for the ASIR in South Asia during this period is forecasted to be the highest (EAPC = 2.49, 95% CI = 1.84, 3.43). Notably, the EAPC for the ASDR and age-standardised DALY rate in South Asia during 2020–2030 is also projected to be the highest among all regions (EAPC of ASDR = 0.56 / 100 000, 95% CI = –0.15 / 100 000, 1.29 / 100 000; EAPC of age-standardised DALY rate = 0.60, 95% CI = 0.18, 1.66 per 100 000 persons) (**Table 1**, Figures S1–S2 in the **Online Supplementary Document**).

For Central Latin America, Eastern sub-Saharan Africa, Oceania, and South Asia, the EAPC for ASIR, ASDR, and age-standardised DALY rate is expected to show an increasing trend between 2020 and 2030. In contrast, the ASDR and age-standardised DALY rates in other regions are generally predicted to decline during this period (**Table 1**, Figure S1 in the **Online Supplementary Document**).

The disease burden of thyroid cancer in 204 countries globally is also forecasted, with a general upward trend expected in the majority of these countries between 2020 and 2030, though a few are predicted to show a declining trend (Table S5 in the **Online Supplementary Document**). The countries with the fastest increasing ASIR are Cabo Verde (EAPC = 3.64, 95% CI = 3.35, 4.21), followed by Egypt (EAPC = 3.44, 95% CI = 0.94, 4.07) and Bangladesh (EAPC = 2.99, 95% CI = 1.98, 3.34). Notably, the countries projected to have the highest EAPC for ASDR and age-standardised DALY rate between 2020–2030 are Cabo Verde (EAPC of ASDR = 5.11, 95% CI = 4.50, 5.39; EAPC of age-standardised DALY rate = 4.00, 95% CI = 2.99, 4.48) and Saint Kitts and Nevis (EAPC of ASDR = 1.90, 95% CI = 1.64, 2.13; EAPC of age-standardised DALY rate = 1.97, 95% CI = 1.44, 2.53) (Tables S5-S6, Figures S6–S8 in the **Online Supplementary Document**).

For the period 2020–2030, South Africa is expected to have the lowest EAPC for both ASDR (−5.46, 95% CI = −5.68, −2.98) and age-standardised DALY rate (−5.13, 95% CI = −7.38, −2.51), while the Republic of Korea is projected to have the lowest EAPC for ASIR (−3.31, 95% CI = 1.87, −0.08) (Tables S5 and S7, Figures S6–S8 in the **Online Supplementary Document**).

### Prediction of global thyroid cancer burden with socio-economic development levels for 2020–2030

From 1990 to 2030, global thyroid cancer burden trends correlate strongly with socio-economic development, showing declining disease indicators in high SDI regions and increasing trends in lower SDI regions. The forecasted trends in disease burden for 2020–2030 showed considerable differences between regions of varying SDI. In regions with high SDI, all three indicators (ASIR, ASDR, and age-standardised DALY rate) are expected to show a declining trend (EAPC of ASIR = −0.37, 95% CI = −1.67, 1.19; EAPC of ASDR = −0.34, 95% CI = −0.26, −0.16; EAPC of age-standardised DALY rate = −0.37, 95 CI = −0.54, −0.36) **(****Table 1****,** Figure S1 in the **Online Supplementary Document****)**. However, for both the low-middle SDI and low SDI regions, an increasing trend is anticipated for all three indicators during 2020 − 2030. Specifically, the growth rate of EAPC for ASIR is highest in the low-middle SDI regions (EAPC = 2.15, 95% CI = 2.05, 2.44), followed by the low SDI regions, where EAPC is 1.73 (95% CI = 1.88, 1.98). However, we have also observed a general trend across regions with different SDI levels between 1990 and 2030, where higher SDI is associated with a higher baseline ASIR (Age-Standardized Incidence Rate) of disease burden (**Table 1****,** Figure S1, panel A in the **Online Supplementary Document**).

We used GAMs to fit the SDI levels of each region from 1990–2019 and thereby forecasted the SDI levels for 2020–2030. During the period from 1990 to 2030, there was a strong positive correlation between SDI and ASIR (r = 0.830, *P* < 0.01). Conversely, there was a negative correlation trend between SDI and ASDR from 1990 to 2030 (r = −0.316, *P* < 0.01). As sociodemographic conditions improved, the mortality rate decreased. This pattern was almost consistent across all regions and over time, with the ASDR decreasing as the SDI increased. Similarly, there was a negative correlation between SDI and age-standardised DALY rate between 1990 and 2030 (r = −0.377, *P* < 0.01) (Figure S10 in the **Online Supplementary Document**).

We conducted a correlation analysis between the EAPC forecasts for 2020–2030 and the SDI levels of various countries in 2019. We found no clear relationship between the EAPC of ASDR and age-standardised DALY rate and the baseline SDI of countries in 2019. As the SDI level increases, the growth rate of disease burden nearly flattens (EAPC of ASDR: r = −0.137, *P* = 0.05; EAPC of age-standardised DALY rate: r = −0.040, *P* = 0.56). However, there is a statistically significant negative correlation between the EAPC of ASIR and the SDI level of countries in 2019 (r = −0.190, *P* = 0.0064). This indicates that, in general, with the increase in SDI, the EAPC of ASIR decreases. On analysing the fitted curves specifically, we found that when the SDI level changes from 0 to 0.6, the EAPC of ASIR continuously increases; however, as the SDI level changes from 0.6 to 1, the EAPC of ASIR consistently decreases (Figure S11 in the **Online Supplementary Document**).

## DISCUSSION

To our knowledge, this is the first study to utilise GAMs for global estimates and predictions on the disease burden of TC. We project that from 2020 to 2030, the global ASIR of TC will annually increase, expecting to reach about 305 078 cases by 2030, approximately 1.3 times the figure in 2019. However, the ASDR is anticipated to decline, while age-standardised DALYs are expected to stay relatively stable. A decrease in age-standardised mortality rates for both genders is foreseen, with a more significant reduction in females. The study shows an increase in TC incidence across all age groups, with an opposite trend observed in the old age group. By 2030, the heaviest TC disease burden is predicted in South Asia. Between 2020 and 2030, SDI is positively correlated with ASIR, but it shows no significant relationship with the growth rates of ASDR or DALYs.

The anticipated decrease in ASDR between 2020 and 2030 is indicative of significant strides in TC treatment [15]. Innovations in targeted therapies and advanced surgical interventions are likely contributing factors to this positive trend, enhancing patient survival rates [16]. Moreover, the decrease in mortality rates may also be attributed to the generally favourable prognosis of most thyroid cancer types, except for anaplastic thyroid cancer [17].

The projection of a stable age-standardised DALY rate for TC from 2020–2030, amidst increasing ASIR and decreasing ASDR, presents a nuanced scenario. This stability likely reflects a balance achieved through early detection, leading to prolonged disease courses but lower mortality rates, and the efficacy of contemporary treatment protocols enabling extended patient survival [18].

The gender-specific analysis in our study uncovers an upward trend in ASIR for both males and females, with the upward trend being greater for females than for males. Gender-related differences in risk factors, such as hormonal influences in women and lifestyle choices in men, likely contribute to these diverging patterns [19]. The projected decline in the ASDR for both sexes indicates advancements in therapeutic approaches and patient survival for thyroid cancer, emphasising the ongoing need to refine health care practices and management strategies [20]. Our study also reveals an increase in thyroid cancer incidence across various age groups, consistent with the notion that the risk of thyroid cancer accumulates over time due to prolonged exposure to risk factors [21]. However, a reduction in incidence rates in older age groups might suggest underdiagnosis or the masking of thyroid cancer by other age-related health issues [22].

Our regional analysis for 2030 points to significant disparities in thyroid cancer burden, with South Asia emerging as a particularly affected area. This trend reflects broader socio-economic and health care shifts, including rapid urbanisation, lifestyle changes, and genetic predispositions, alongside enhanced detection and reporting capabilities [8]. However, it should not be overlooked that overdiagnosis in the Asia-Pacific region may also be responsible for a significantly higher burden of disease than in other regions [23]. Meanwhile, upward trends in regions like Central Latin America, Eastern sub-Saharan Africa, and Oceania suggest a shift in the global epidemiology of thyroid cancer. In contrast, regions such as South Africa and countries like the Republic of Korea show declining disease rates, likely due to effective public health policies and environmental management strategies [2].

The dynamic relationship between the global thyroid cancer burden and socioeconomic development from 2020 to 2030 shows that while high SDI regions are seeing declines in ASIR, ASDR, and DALY rates due to medical and health care advancements [24], low to middle SDI regions face increases in these indicators due to transitions from infectious diseases to chronic conditions like cancer [25]. Our analysis between the EAPC and SDI levels in 2019 indicates a positive correlation between SDI and ASIR, while no clear relationship is observed with ASDR and DALY rate growths. This suggests that other factors, such as health care policies and public health initiatives, play a significant role beyond mere economic development [26]. Multiple epidemiological studies have shown that lower SDI regions are associated with an increased risk of thyroid cancer burden [27]. Previous research indicates that iodine deficiency [28] and insufficient protection against ionizing radiation [29] in middle and low SDI regions elevate thyroid cancer risk, although the exact biological mechanisms require further investigation. This may also contribute to the rising disease burden of thyroid cancer from 2020 to 2030. Meanwhile, the decrease in EAPC for ASIR with rising SDI levels, especially beyond an SDI of 0.6, hints at a potential saturation point in higher SDI regions, implying a limit to further improvements achievable with increased development and health care efficiency. This observation underscores the importance of tailoring health care strategies and interventions to the unique socio-economic contexts of different regions [7].

Our study uses the Global Burden of Disease database for an extensive analysis of thyroid cancer, but it has limitations. The GBD data, while extensive, faces accuracy and completeness issues due to varied reporting systems and data collection methods worldwide [30]. Disparities in diagnostic opportunities across regions, affecting thyroid cancer detection and reporting, may compromise data comparability by leading to variations in case identification and diagnosis stages. The robustness of generalised additive models relies on historical data stability and assumptions, which may not predict future trends or disruptions accurately [31]. Our analysis of thyroid cancer burden across SDI regions, although insightful, might not fully reflect the intricate sociocultural and economic factors of these areas. Additionally, the study may overlook factors like genetic predispositions, environmental pollution, and lifestyle changes affecting thyroid cancer burden. Finally, because the data used in the study are summary rather than individual and lack detailed dynamic variables, mechanistic analysis driving the observed trend cannot be conducted.

Our study underscores the necessity of tailored strategies for thyroid cancer prevention, early detection, and treatment, especially in high-risk regions and populations. It stresses the role of environmental and lifestyle factors in thyroid cancer risk and calls for specialised medical and public health approaches. The study, while offering a thorough global overview of thyroid cancer, also highlights the ongoing need for detailed research to effectively address the unique challenges of thyroid cancer in diverse global contexts. This work provides valuable insights and identifies future research and intervention areas. Our research emphasises the need to integrate environmental, genetic, and socio-economic factors in future studies for a more comprehensive assessment of thyroid cancer's disease burden through individual exposure analysis across various regions and countries.

## CONCLUSIONS

This study predicts changing global trends in TC burden from 2020 to 2030, highlighting regional and gender-specific variations. The increasing ASIR, particularly in South Asia and Central Latin America, underscores the influence of socioeconomic factors and health care access. Meanwhile, the decreasing ASDR suggests advancements in TC management. The study emphasises the need for targeted prevention and tailored health care strategies, especially in developed regions with higher incidence rates. It advocates for comprehensive cancer registries to guide public health policies and reduce TC-related health disparities worldwide.

## Additional material


Online Supplementary Document
