# Supplementary material for: Assessing and projecting the global burden of thyroid cancer, 1990–2030: Analysis of the Global Burden of Disease Study
Source: J Glob Health. 2024 Apr 5;14:04090. doi: 10.7189/jogh.14.04090 (PMC10995745; doi:10.7189/jogh.14.04090)

Supplementary materials

Text 1 The SDI groups of different countries estimated in GBD 2019.

Low SDI	Afghanistan, Benin, Burkina Faso, Burundi, Central African Republic, Chad, Comoros, Côte d'Ivoire, DR Congo, Eritrea, Ethiopia, The Gambia, Guinea, Guinea-Bissau, Haiti, Liberia, Madagascar, Malawi, Mali, Mozambique, Nepal, Niger, Pakistan, Papua New Guinea, Rwanda, Senegal, Sierra Leone, Solomon Islands, Somalia, South Sudan, Tanzania, Togo, Uganda, Yemen
Low-middle SDI	Angola, Bangladesh, Belize, Bhutan, Bolivia (Plurinational State of), Cape Verde, Cambodia, Cameroon, Congo (Brazzaville), Djibouti, Dominican Republic, ElSalvador, Eswatini, Federated States of Micronesia, Ghana, Guatemala, Honduras, India, Kenya, Kiribati, Kyrgyzstan, Laos, Lesotho, Maldives, Marshall Islands, Mauritania, Mongolia, Morocco, Myanmar, Nicaragua, Nigeria, North Korea, Palestine, São Tomé and Príncipe, Sudan, Tajikistan, Timor-Leste, Tuvalu, Vanuatu, Venezuela (Bolivarian Republic of), Zambia, Zimbabwe
Middle SDI	Albania, Algeria, Armenia, Azerbaijan, Botswana, Brazil, China, Colombia, Costa Rica, Cuba, Ecuador, Egypt, Equatorial Guinea, Fiji, Gabon, Grenada, Guyana, Indonesia, Iran (Islamic Republic of), Iraq, Jamaica, Mexico, Namibia,Nauru, Panama, Paraguay, Peru, Philippines, Saint Lucia, Saint Vincent and the Grenadines, Samoa, South Africa, Suriname, Syrian Arab Republic, Thailand,Tokelau, Tonga, Tunisia, Turkmenistan, Uzbekistan, Vietnam
High-middle SDI	American Samoa, Antigua and Barbuda, Argentina, Bahamas, Bahrain, Barbados, Belarus, Bosnia and Herzegovina, Bulgaria, Chile, Cook Islands, Croatia, Dominica, Georgia, Greece, Greenland, Hungary, Israel, Italy, Jordan, Kazakhstan, Lebanon, Libya, Malaysia, Malta, Mauritius, Montenegro, Niue, North Macedonia, Northern Mariana Islands, Oman, Palau, Poland, Portugal, Republic of Moldova, Romania, Russia, Saint Kitts and Nevis, Serbia, Seychelles,Spain, Sri Lanka, Trinidad and Tobago, Turkey, Ukraine, United States Virgin Islands, Uruguay
High SDI	Andorra, Australia, Austria, Belgium, Bermuda, Brunei Darussalam, Canada,Cyprus, Czechia, Denmark, Estonia, Finland, France, Germany, Guam, Iceland, Ireland, Japan, Kuwait, Latvia, Lithuania, Luxembourg, Monaco, Netherlands, New Zealand, Norway, Puerto Rico, Qatar, San Marino, Saudi Arabia, Singapore, Slovakia, Slovenia, South Korea, Sweden, Switzerland, Taiwan (Province of China), United Arab Emirates, United Kingdom, United States of America

Table S1. The Numbers and age-standardized Rate of global Thyroid cancer Cases, Deaths, and Disability-Adjusted Life Years Between 1990 and 2030.

	Number			Rate		
	Incidence	Deaths	DALYs (Disability-Adjusted Life Years)	Incidence	Deaths	DALYs (Disability-Adjusted Life Years)
1990	87582.64 (82236.09, 92717.22)	22966.38 (21553.76, 25227.70)	667462.46 (613562.63, 732753.11)	2.01 (1.90, 2.12)	0.60 (0.56, 0.66)	15.55 (14.40, 17.02)
1991	90868.51 (85539.46, 95966.70)	23427.07 (21982.80, 25813.30)	680478.88 (629081.33, 743255.21)	2.04 (1.93, 2.15)	0.60 (0.56, 0.66)	15.52 (14.44, 16.93)
1992	94349.60 (89051.58, 100088.88)	24000.31 (22554.42, 26261.61)	696734.81 (639253.22, 758148.59)	2.07 (1.97, 2.19)	0.60 (0.56, 0.66)	15.56 (14.39, 16.87)
1993	98974.78 (92824.09, 104517.57)	24656.66 (23170.82, 27111.76)	715013.07 (658607.91, 777586.97)	2.13 (2.01, 2.25)	0.60 (0.56, 0.66)	15.64 (14.54, 17.01)
1994	103016.82 (97026.95, 108764.16)	25239.44 (23759.35, 27559.43)	733887.30 (678044.17, 791948.58)	2.17 (2.05, 2.28)	0.60 (0.56, 0.66)	15.71 (14.61, 16.94)
1995	106814.68 (100108.98, 112493.56)	25739.26 (24179.73, 28238.32)	748649.10 (688271.79, 810741.85)	2.17 (2.05, 2.28)	0.60 (0.56, 0.66)	15.69 (14.54, 16.97)
1996	110373.89 (103099.90, 115618.19)	26205.49 (24603.16, 28448.91)	761624.31 (701319.82, 819266.34)	2.22 (2.09, 2.32)	0.60 (0.56, 0.65)	15.62 (14.46, 16.75)
1997	114216.38 (106700.97, 119707.95)	26754.02 (25027.47, 28927.56)	777475.88 (713059.01, 838486.90)	2.22 (2.09, 2.32)	0.59 (0.56, 0.64)	15.60 (14.41, 16.78)
1998	118717.95 (109903.89, 124083.50)	27348.04 (25658.69, 29560.15)	794553.88 (727997.46, 853611.00)	2.28 (2.13, 2.39)	0.59 (0.55, 0.64)	15.59 (14.41, 16.67)
1999	123615.89 (114366.29, 129425.31)	28006.03 (26106.40, 30168.01)	813865.89 (744574.59, 877276.41)	2.32 (2.16, 2.43)	0.59 (0.55, 0.64)	15.62 (14.32, 16.78)
2000	128373.57 (118473.44, 133942.40)	28649.81 (26704.34, 30754.16)	832310.05 (756969.16, 892950.88)	2.36 (2.19, 2.45)	0.59 (0.55, 0.64)	15.63 (14.34, 16.75)
2001	132907.34 (122884.63, 138642.90)	29260.05 (27227.97, 31064.31)	846573.90 (772921.95, 908763.66)	2.38 (2.21, 2.48)	0.59 (0.55, 0.63)	15.56 (14.30, 16.61)
2002	138024.21 (126881.29, 143694.08)	30034.74 (27986.04, 32048.41)	863435.93 (795217.69, 922284.42)	2.42 (2.25, 2.52)	0.59 (0.55, 0.63)	15.54 (14.38, 16.60)
2003	143845.46 (133126.01, 149359.02)	30752.82 (28584.89, 32565.90)	879889.29 (808752.41, 934954.02)	2.47 (2.30, 2.56)	0.59 (0.55, 0.63)	15.51 (14.34, 16.47)
2004	149202.93 (137596.51, 154827.13)	31340.76 (28913.12, 33033.60)	894919.87 (821622.67, 949085.31)	2.51 (2.32, 2.60)	0.59 (0.54, 0.62)	15.41 (14.19, 16.32)
2005	156587.39 (143999.63, 162674.21)	32335.24 (29832.66, 34110.80)	921032.51 (843720.27, 977147.33)	2.57 (2.38, 2.67)	0.59 (0.54, 0.62)	15.51 (14.27, 16.45)
2006	162485.34 (148319.64, 168266.15)	33227.47 (30593.70, 34889.44)	942956.03 (864543.11, 1001806.08)	2.60 (2.39, 2.70)	0.59 (0.54, 0.62)	15.51 (14.27, 16.46)
2007	169570.83 (154543.98, 175388.09)	34135.39 (31420.80, 35756.01)	966149.69 (876182.96, 1021793.86)	2.66 (2.43, 2.75)	0.59 (0.54, 0.62)	15.52 (14.04, 16.39)
2008	177860.60 (160723.31, 184408.21)	35272.35 (32399.72, 36943.38)	995649.27 (902046.75, 1053492.95)	2.72 (2.47, 2.82)	0.59 (0.54, 0.62)	15.62 (14.17, 16.50)
2009	184899.42 (167042.97, 191581.82)	36165.87 (32705.62, 37813.21)	1018104.64 (918725.84, 1075365.67)	2.77 (2.50, 2.87)	0.59 (0.54, 0.62)	15.60 (14.07, 16.47)

2010	191196.69 (171482.61, 197887.65)	37268.46 (33797.07, 38957.16)	1045884.73 (943435.92, 1104348.79)	2.80 (2.51, 2.90)	0.60 (0.54, 0.62)	15.65 (14.12, 16.52)
2011	195015.54 (174980.81, 202216.57)	38122.42 (34369.36, 39927.21)	1063260.31 (956226.78, 1125106.93)	2.79 (2.51, 2.90)	0.59 (0.53, 0.62)	15.55 (14.04, 16.43)
2012	197785.88 (179540.67, 205496.71)	38953.17 (35097.71, 40766.76)	1078502.20 (974830.56, 1137597.40)	2.77 (2.52, 2.88)	0.59 (0.53, 0.62)	15.41 (13.91, 16.25)
2013	201491.96 (183510.55, 209770.17)	39786.67 (35778.40, 41571.29)	1095933.08 (993764.21, 1157415.61)	2.76 (2.52, 2.87)	0.59 (0.52, 0.61)	15.29 (13.87, 16.14)
2014	204779.30 (188291.73, 213511.10)	40408.70 (36586.70, 42295.89)	1107138.48 (1006137.21, 1172619.39)	2.75 (2.53, 2.87)	0.58 (0.52, 0.61)	15.09 (13.72, 15.99)
2015	209736.52 (192877.57, 219422.35)	41382.62 (37546.22, 43401.85)	1130453.94 (1029920.08, 1194119.61)	2.76 (2.53, 2.88)	0.58 (0.52, 0.61)	15.06 (13.71, 15.90)
2016	214507.60 (197092.64, 225525.93)	42347.45 (38319.82, 44568.43)	1153595.25 (1044065.34, 1225839.08)	2.76 (2.53, 2.90)	0.57 (0.52, 0.60)	15.02 (13.63, 15.95)
2017	219579.31 (201634.82, 232966.60)	43210.23 (38898.25, 45699.88)	1174492.68 (1065227.16, 1254514.56)	2.77 (2.54, 2.93)	0.57 (0.51, 0.60)	14.94 (13.54, 15.97)
2018	227155.35 (208277.92, 243443.55)	44374.57 (40273.76, 47287.51)	1203523.46 (1091964.81, 1299337.41)	2.80 (2.57, 3.00)	0.57 (0.51, 0.61)	14.97 (13.61, 16.15)
2019	233846.64 (211636.89, 252806.55)	45575.96 (41289.61, 48775.34)	1231841.05 (1113585.39, 1327064.40)	2.83 (2.56, 3.06)	0.57 (0.51, 0.61)	14.98 (13.55, 16.14)
2020	239659.38 (216903.21, 260811.76)	46559.44 (42217.58, 50058.29)	1255983.97 (1135172.50, 1361508.63)	2.85 (2.57, 3.10)	0.57 (0.51, 0.61)	14.95 (13.52, 16.21)
2021	246201.25 (221796.86, 269959.90)	47655.30 (43242.37, 51486.03)	1282776.55 (1157956.27, 1396446.94)	2.87 (2.58, 3.15)	0.56 (0.51, 0.61)	14.95 (13.50, 16.29)
2022	252743.12 (226690.52, 279108.04)	48751.16 (44267.16, 52913.77)	1309569.14 (1180740.03, 1431385.25)	2.90 (2.59, 3.21)	0.56 (0.51, 0.61)	14.95 (13.48, 16.37)
2023	259284.99 (231584.17, 288256.18)	49847.01 (45291.95, 54341.51)	1336361.72 (1203523.80, 1466323.55)	2.92 (2.60, 3.26)	0.56 (0.51, 0.61)	14.95 (13.46, 16.45)
2024	265826.86 (236477.82, 297404.32)	50942.87 (46316.74, 55769.25)	1363154.30 (1226307.57, 1501261.86)	2.95 (2.60, 3.32)	0.56 (0.51, 0.62)	14.95 (13.44, 16.52)
2025	272368.73 (241371.48, 306552.46)	52038.73 (47341.52, 57196.99)	1389946.89 (1249091.33, 1536200.17)	2.97 (2.61, 3.37)	0.56 (0.51, 0.62)	14.95 (13.42, 16.60)
2026	278910.60 (246265.13, 315700.59)	53134.59 (48366.31, 58624.73)	1416739.47 (1271875.10, 1571138.48)	3.00 (2.62, 3.43)	0.56 (0.51, 0.62)	14.95 (13.40, 16.68)
2027	285452.47 (251158.79, 324848.73)	54230.44 (49391.10, 60052.47)	1443532.05 (1294658.87, 1606076.78)	3.02 (2.63, 3.48)	0.55 (0.50, 0.62)	14.95 (13.38, 16.75)
2028	291994.34 (256052.44, 333996.87)	55326.30 (50415.89, 61480.21)	1470324.64 (1317442.64, 1641015.09)	3.05 (2.64, 3.53)	0.55 (0.50, 0.62)	14.95 (13.36, 16.83)
2029	298536.21 (260946.09, 343145.01)	56422.16 (51440.68, 62907.95)	1497117.22 (1340226.40, 1675953.40)	3.07 (2.65, 3.59)	0.55 (0.50, 0.62)	14.95 (13.34, 16.91)
2030	305078.08 (265839.75, 352293.15)	57518.02 (52465.47, 64335.68)	1523909.81 (1363010.17, 1710891.71)	3.09 (2.65, 3.64)	0.55 (0.50, 0.63)	14.95 (13.32, 16.98)

Table S2. The Incidence Rate of global Thyroid cancer burden Between 1990 and 2030, by ages.

incidence	10-14 years	15-19 years	20-24 years	25-29 years	30-34 years	35-39 years	40-44 years	45-49 years	50-54 years	55-59 years	60-64 years	65-69 years	70-74 years	75-79 years	80-84	85-89	90-94	95+ years
1990	0.14 (0.12, 0.16)	0.29 (0.24, 0.33)	0.50 (0.40, 0.57)	0.84 (0.68, 0.95)	1.36 (1.15, 1.52)	1.89 (1.68, 2.08)	2.67 (2.45, 2.89)	3.38 (3.15, 3.61)	4.45 (4.17, 4.73)	5.18 (4.89, 5.49)	5.81 (5.53, 6.19)	6.46 (6.15, 6.95)	6.76 (6.44, 7.43)	7.72 (7.27, 8.47)	7.45 (6.69, 8.16)	7.87 (6.83, 8.61)	5.72 (4.67, 6.44)	8.29 (6.42, 9.37)
1991	0.14 (0.12, 0.16)	0.29 (0.25, 0.34)	0.51 (0.41, 0.58)	0.84 (0.69, 0.96)	1.40 (1.18, 1.55)	1.93 (1.72, 2.12)	2.75 (2.52, 2.96)	3.43 (3.20, 3.65)	4.53 (4.24, 4.80)	5.23 (4.95, 5.55)	5.85 (5.58, 6.25)	6.51 (6.18, 7.01)	6.89 (6.56, 7.59)	7.75 (7.29, 8.54)	7.51 (6.72, 8.18)	7.84 (6.79, 8.60)	5.68 (4.64, 6.36)	8.34 (6.44, 9.43)
1992	0.14 (0.12, 0.16)	0.30 (0.25, 0.35)	0.52 (0.41, 0.60)	0.85 (0.70, 0.97)	1.43 (1.21, 1.59)	1.97 (1.75, 2.16)	2.81 (2.57, 3.02)	3.52 (3.29, 3.74)	4.62 (4.33, 4.89)	5.29 (5.00, 5.62)	5.90 (5.62, 6.29)	6.57 (6.25, 7.06)	7.06 (6.71, 7.71)	7.76 (7.29, 8.56)	7.60 (6.80, 8.30)	7.94 (6.85, 8.73)	5.76 (4.69, 6.43)	8.55 (6.56, 9.66)
1993	0.15 (0.13, 0.16)	0.31 (0.26, 0.35)	0.53 (0.43, 0.61)	0.87 (0.72, 0.99)	1.47 (1.25, 1.63)	2.05 (1.84, 2.24)	2.90 (2.64, 3.11)	3.65 (3.42, 3.87)	4.77 (4.48, 5.04)	5.44 (5.15, 5.78)	5.99 (5.69, 6.41)	6.69 (6.38, 7.21)	7.29 (6.92, 7.97)	7.83 (7.37, 8.64)	7.75 (6.94, 8.42)	8.13 (7.01, 8.93)	5.87 (4.79, 6.55)	8.89 (6.79, 10.09)
1994	0.15 (0.13, 0.17)	0.31 (0.27, 0.35)	0.56 (0.44, 0.64)	0.92 (0.75, 1.03)	1.54 (1.29, 1.69)	2.12 (1.90, 2.30)	2.96 (2.68, 3.18)	3.79 (3.54, 4.02)	4.85 (4.54, 5.12)	5.51 (5.23, 5.81)	5.99 (5.70, 6.38)	6.70 (6.38, 7.21)	7.41 (7.04, 8.09)	7.76 (7.28, 8.60)	7.78 (6.95, 8.46)	8.20 (7.07, 8.97)	5.89 (4.79, 6.56)	9.08 (6.92, 10.33)
1995	0.16 (0.13, 0.17)	0.32 (0.28, 0.36)	0.58 (0.45, 0.66)	0.97 (0.78, 1.09)	1.59 (1.32, 1.77)	2.17 (1.95, 2.35)	2.98 (2.70, 3.20)	3.93 (3.67, 4.19)	4.88 (4.58, 5.14)	5.55 (5.27, 5.86)	5.97 (5.69, 6.36)	6.73 (6.41, 7.24)	7.45 (7.08, 8.13)	7.82 (7.33, 8.62)	7.82 (6.98, 8.49)	8.31 (7.16, 9.08)	5.95 (4.83, 6.63)	9.30 (7.07, 10.60)
1996	0.16 (0.14, 0.18)	0.32 (0.28, 0.37)	0.60 (0.47, 0.69)	1.00 (0.80, 1.13)	1.63 (1.32, 1.79)	2.20 (1.97, 2.39)	3.01 (2.73, 3.23)	4.01 (3.73, 4.25)	4.92 (4.61, 5.20)	5.61 (5.33, 5.88)	5.96 (5.69, 6.34)	6.75 (6.44, 7.24)	7.42 (7.07, 8.06)	7.90 (7.41, 8.65)	7.82 (6.95, 8.48)	8.38 (7.20, 9.11)	5.97 (4.84, 6.63)	9.48 (7.17, 10.82)
1997	0.16 (0.14, 0.18)	0.33 (0.29, 0.37)	0.62 (0.49, 0.71)	1.04 (0.82, 1.17)	1.67 (1.36, 1.84)	2.25 (2.01, 2.45)	3.08 (2.77, 3.29)	4.03 (3.75, 4.27)	4.98 (4.67, 5.24)	5.65 (5.37, 5.93)	5.99 (5.72, 6.37)	6.76 (6.45, 7.25)	7.41 (7.05, 8.02)	8.03 (7.53, 8.75)	7.81 (6.93, 8.46)	8.44 (7.23, 9.19)	6.01 (4.88, 6.67)	9.68 (7.29, 11.02)
1998	0.17 (0.14, 0.18)	0.34 (0.29, 0.38)	0.65 (0.51, 0.75)	1.08 (0.84, 1.23)	1.72 (1.38, 1.90)	2.29 (2.03, 2.47)	3.16 (2.83, 3.37)	4.05 (3.75, 4.28)	5.05 (4.73, 5.32)	5.73 (5.46, 6.02)	6.07 (5.80, 6.45)	6.83 (6.51, 7.28)	7.46 (7.11, 8.04)	8.22 (7.70, 8.91)	7.82 (6.94, 8.47)	8.54 (7.35, 9.31)	6.05 (4.91, 6.70)	9.85 (7.43, 11.23)
1999	0.17 (0.15, 0.19)	0.34 (0.30, 0.39)	0.67 (0.52, 0.77)	1.12 (0.87, 1.28)	1.76 (1.41, 1.94)	2.33 (2.06, 2.52)	3.24 (2.92, 3.45)	4.12 (3.81, 4.36)	5.17 (4.83, 5.43)	5.88 (5.57, 6.16)	6.17 (5.87, 6.50)	6.86 (6.52, 7.27)	7.52 (7.16, 8.08)	8.39 (7.82, 9.03)	7.77 (6.86, 8.40)	8.64 (7.40, 9.41)	6.14 (4.96, 6.83)	10.07 (7.60, 11.47)
2000	0.17 (0.15, 0.19)	0.35 (0.30, 0.39)	0.69 (0.53, 0.79)	1.15 (0.89, 1.32)	1.79 (1.44, 1.97)	2.38 (2.09, 2.57)	3.32 (2.99, 3.52)	4.18 (3.85, 4.41)	5.34 (4.97, 5.60)	5.96 (5.63, 6.24)	6.25 (5.95, 6.57)	6.90 (6.56, 7.29)	7.56 (7.19, 8.12)	8.41 (7.84, 9.01)	7.74 (6.85, 8.34)	8.62 (7.38, 9.36)	6.12 (4.92, 6.79)	10.17 (7.66, 11.57)
2001	0.17 (0.15, 0.19)	0.35 (0.30, 0.39)	0.69 (0.55, 0.79)	1.17 (0.91, 1.32)	1.81 (1.49, 1.99)	2.41 (2.12, 2.59)	3.38 (3.04, 3.58)	4.25 (3.89, 4.49)	5.47 (5.10, 5.74)	6.02 (5.65, 6.30)	6.34 (6.04, 6.67)	6.95 (6.59, 7.34)	7.63 (7.22, 8.11)	8.45 (7.85, 9.02)	7.78 (6.85, 8.35)	8.61 (7.32, 9.37)	6.16 (4.95, 6.83)	10.28 (7.76, 11.69)
2002	0.16 (0.15, 0.18)	0.35 (0.30, 0.39)	0.69 (0.56, 0.79)	1.16 (0.92, 1.30)	1.78 (1.49, 1.94)	2.43 (2.14, 2.64)	3.47 (3.12, 3.68)	4.34 (3.98, 4.59)	5.57 (5.19, 5.85)	6.25 (5.85, 6.55)	6.53 (6.21, 6.83)	7.07 (6.68, 7.45)	7.73 (7.30, 8.20)	8.55 (7.90, 9.08)	7.91 (6.94, 8.48)	8.64 (7.36, 9.44)	6.27 (5.02, 6.95)	10.71 (8.07, 12.18)
2003	0.16 (0.15, 0.17)	0.35 (0.31, 0.39)	0.69 (0.57, 0.78)	1.15 (0.93, 1.27)	1.79 (1.50, 1.94)	2.46 (2.19, 2.65)	3.54 (3.19, 3.76)	4.46 (4.09, 4.70)	5.68 (5.30, 5.98)	6.47 (6.04, 6.77)	6.75 (6.36, 7.07)	7.26 (6.83, 7.62)	7.85 (7.40, 8.28)	8.65 (8.01, 9.15)	8.07 (7.08, 8.62)	8.66 (7.36, 9.49)	6.30 (5.04, 7.00)	11.14 (8.34, 12.70)

2004	0.16 (0.15, 0.18)	0.36 (0.32, 0.40)	0.70 (0.58, 0.79)	1.18 (0.96, 1.31)	1.83 (1.55, 1.98)	2.51 (2.23, 2.69)	3.59 (3.23, 3.81)	4.58 (4.21, 4.83)	5.70 (5.31, 6.01)	6.56 (6.12, 6.87)	6.87 (6.47, 7.19)	7.36 (6.94, 7.71)	7.87 (7.37, 8.31)	8.68 (8.01, 9.13)	8.18 (7.14, 8.73)	8.57 (7.20, 9.34)	6.33 (5.04, 7.04)	11.70 (8.72, 13.34)
2005	0.17 (0.15, 0.18)	0.37 (0.32, 0.40)	0.72 (0.59, 0.81)	1.22 (0.99, 1.36)	1.88 (1.58, 2.03)	2.59 (2.28, 2.79)	3.66 (3.28, 3.89)	4.75 (4.31, 5.00)	5.82 (5.42, 6.12)	6.81 (6.34, 7.14)	7.02 (6.58, 7.35)	7.54 (7.11, 7.86)	8.00 (7.49, 8.42)	8.83 (8.10, 9.24)	8.31 (7.24, 8.85)	8.65 (7.21, 9.43)	6.42 (5.10, 7.14)	12.32 (9.15, 14.07)
2006	0.17 (0.15, 0.18)	0.37 (0.33, 0.41)	0.73 (0.60, 0.81)	1.25 (1.02, 1.38)	1.92 (1.62, 2.08)	2.66 (2.32, 2.86)	3.73 (3.32, 3.99)	4.80 (4.33, 5.07)	5.87 (5.44, 6.17)	6.93 (6.41, 7.26)	7.05 (6.50, 7.37)	7.65 (7.12, 7.97)	8.07 (7.55, 8.48)	8.94 (8.15, 9.35)	8.38 (7.26, 8.91)	8.72 (7.25, 9.53)	6.47 (5.10, 7.23)	12.69 (9.38, 14.55)
2007	0.17 (0.15, 0.18)	0.37 (0.33, 0.41)	0.74 (0.62, 0.83)	1.29 (1.04, 1.42)	1.99 (1.67, 2.15)	2.74 (2.37, 2.94)	3.84 (3.41, 4.10)	4.88 (4.40, 5.15)	5.96 (5.53, 6.24)	7.01 (6.47, 7.32)	7.21 (6.66, 7.54)	7.82 (7.25, 8.15)	8.17 (7.62, 8.57)	9.07 (8.24, 9.50)	8.45 (7.31, 8.99)	8.87 (7.37, 9.73)	6.51 (5.10, 7.29)	13.07 (9.56, 15.04)
2008	0.17 (0.16, 0.19)	0.38 (0.33, 0.42)	0.75 (0.63, 0.84)	1.33 (1.07, 1.47)	2.07 (1.72, 2.25)	2.83 (2.41, 3.06)	3.97 (3.45, 4.26)	4.99 (4.50, 5.25)	6.12 (5.66, 6.43)	7.09 (6.53, 7.42)	7.38 (6.79, 7.69)	8.01 (7.41, 8.35)	8.34 (7.71, 8.75)	9.26 (8.38, 9.71)	8.64 (7.44, 9.23)	9.10 (7.48, 10.00)	6.61 (5.15, 7.41)	13.40 (9.81, 15.49)
2009	0.18 (0.16, 0.20)	0.38 (0.34, 0.42)	0.76 (0.64, 0.85)	1.37 (1.10, 1.52)	2.14 (1.77, 2.34)	2.90 (2.45, 3.13)	4.03 (3.47, 4.32)	5.06 (4.59, 5.34)	6.24 (5.76, 6.53)	7.11 (6.57, 7.44)	7.49 (6.96, 7.82)	8.19 (7.50, 8.53)	8.45 (7.78, 8.86)	9.36 (8.42, 9.82)	8.71 (7.48, 9.32)	9.23 (7.56, 10.13)	6.60 (5.12, 7.41)	13.57 (9.83, 15.69)
2010	0.18 (0.17, 0.20)	0.39 (0.35, 0.43)	0.78 (0.66, 0.87)	1.40 (1.13, 1.55)	2.20 (1.83, 2.40)	2.95 (2.49, 3.19)	4.08 (3.51, 4.37)	5.06 (4.59, 5.33)	6.30 (5.81, 6.59)	7.11 (6.62, 7.45)	7.62 (7.07, 7.96)	8.29 (7.59, 8.65)	8.52 (7.82, 8.90)	9.43 (8.45, 9.92)	8.79 (7.55, 9.40)	9.30 (7.60, 10.19)	6.69 (5.20, 7.52)	13.88 (10.02, 16.05)
2011	0.18 (0.17, 0.20)	0.39 (0.35, 0.43)	0.78 (0.65, 0.87)	1.38 (1.13, 1.53)	2.20 (1.83, 2.40)	2.92 (2.47, 3.15)	4.08 (3.47, 4.38)	4.98 (4.54, 5.27)	6.30 (5.82, 6.63)	7.14 (6.63, 7.46)	7.68 (7.10, 8.01)	8.23 (7.52, 8.62)	8.52 (7.82, 8.93)	9.44 (8.41, 9.93)	8.82 (7.56, 9.47)	9.38 (7.64, 10.28)	6.79 (5.28, 7.62)	14.30 (10.31, 16.50)
2012	0.18 (0.17, 0.20)	0.39 (0.35, 0.43)	0.76 (0.65, 0.86)	1.34 (1.11, 1.50)	2.16 (1.81, 2.37)	2.88 (2.48, 3.09)	4.05 (3.47, 4.35)	4.90 (4.46, 5.18)	6.27 (5.79, 6.59)	7.17 (6.62, 7.51)	7.61 (7.07, 7.94)	8.19 (7.52, 8.56)	8.51 (7.85, 8.92)	9.40 (8.33, 9.91)	8.79 (7.43, 9.42)	9.37 (7.62, 10.28)	6.84 (5.33, 7.68)	14.47 (10.30, 16.74)
2013	0.18 (0.17, 0.20)	0.39 (0.35, 0.43)	0.75 (0.64, 0.85)	1.33 (1.11, 1.48)	2.16 (1.80, 2.37)	2.88 (2.52, 3.10)	4.05 (3.51, 4.33)	4.85 (4.45, 5.14)	6.25 (5.78, 6.57)	7.19 (6.65, 7.59)	7.53 (7.03, 7.88)	8.14 (7.55, 8.51)	8.49 (7.83, 8.91)	9.37 (8.38, 9.91)	8.80 (7.47, 9.45)	9.41 (7.59, 10.31)	6.90 (5.39, 7.75)	14.73 (10.55, 17.04)
2014	0.18 (0.17, 0.20)	0.39 (0.34, 0.43)	0.74 (0.64, 0.84)	1.30 (1.11, 1.46)	2.14 (1.82, 2.36)	2.88 (2.53, 3.12)	4.05 (3.53, 4.36)	4.79 (4.40, 5.08)	6.23 (5.77, 6.56)	7.23 (6.71, 7.63)	7.48 (6.96, 7.83)	8.05 (7.49, 8.44)	8.40 (7.74, 8.87)	9.32 (8.33, 9.84)	8.77 (7.43, 9.44)	9.33 (7.51, 10.25)	6.88 (5.36, 7.73)	14.92 (10.66, 17.29)
2015	0.18 (0.17, 0.20)	0.38 (0.34, 0.43)	0.74 (0.63, 0.85)	1.29 (1.08, 1.45)	2.14 (1.85, 2.36)	2.92 (2.57, 3.16)	4.05 (3.57, 4.38)	4.79 (4.40, 5.09)	6.25 (5.79, 6.62)	7.33 (6.78, 7.72)	7.47 (6.97, 7.85)	8.13 (7.57, 8.55)	8.39 (7.77, 8.87)	9.29 (8.29, 9.82)	8.70 (7.37, 9.35)	9.29 (7.47, 10.21)	6.86 (5.34, 7.71)	15.15 (10.81, 17.54)
2016	0.18 (0.17, 0.20)	0.38 (0.34, 0.44)	0.76 (0.65, 0.86)	1.30 (1.10, 1.48)	2.16 (1.86, 2.39)	2.95 (2.60, 3.20)	4.06 (3.55, 4.38)	4.79 (4.42, 5.11)	6.21 (5.75, 6.62)	7.36 (6.85, 7.76)	7.50 (6.97, 7.91)	8.15 (7.58, 8.56)	8.36 (7.68, 8.86)	9.24 (8.25, 9.82)	8.62 (7.34, 9.32)	9.22 (7.42, 10.14)	6.83 (5.28, 7.69)	15.32 (10.89, 17.80)
2017	0.18 (0.16, 0.20)	0.39 (0.34, 0.45)	0.78 (0.66, 0.90)	1.32 (1.12, 1.52)	2.19 (1.90, 2.44)	2.99 (2.63, 3.27)	4.11 (3.63, 4.48)	4.81 (4.39, 5.18)	6.19 (5.69, 6.69)	7.33 (6.75, 7.84)	7.52 (6.98, 8.01)	8.06 (7.49, 8.61)	8.37 (7.67, 8.95)	9.18 (8.20, 9.83)	8.53 (7.24, 9.25)	9.08 (7.28, 10.03)	6.69 (5.17, 7.57)	15.06 (10.55, 17.60)
2018	0.19 (0.17, 0.21)	0.41 (0.35, 0.47)	0.82 (0.69, 0.95)	1.38 (1.17, 1.61)	2.25 (1.97, 2.54)	3.06 (2.70, 3.37)	4.18 (3.72, 4.60)	4.87 (4.38, 5.31)	6.25 (5.67, 6.80)	7.34 (6.73, 7.90)	7.64 (7.03, 8.24)	8.05 (7.49, 8.68)	8.45 (7.76, 9.12)	9.27 (8.31, 9.99)	8.57 (7.27, 9.34)	9.08 (7.24, 10.12)	6.65 (5.10, 7.57)	14.98 (10.40, 17.56)
2019	0.19 (0.17, 0.21)	0.41 (0.36, 0.48)	0.84 (0.70, 0.99)	1.41 (1.19, 1.65)	2.29 (1.98, 2.60)	3.12 (2.74, 3.46)	4.23 (3.71, 4.67)	4.93 (4.43, 5.42)	6.29 (5.68, 6.93)	7.33 (6.67, 8.02)	7.72 (7.07, 8.40)	8.07 (7.42, 8.75)	8.49 (7.70, 9.22)	9.35 (8.36, 10.10)	8.56 (7.27, 9.35)	9.10 (7.35, 10.16)	6.63 (5.09, 7.56)	14.91 (10.38, 17.56)

2020	0.19 (0.17, 0.21)	0.42 (0.36, 0.49)	0.87 (0.72, 1.02)	1.44 (1.22, 1.70)	2.33 (2.02, 2.66)	3.17 (2.78, 3.54)	4.27 (3.77, 4.75)	4.96 (4.42, 5.49)	6.29 (5.64, 6.99)	7.34 (6.65, 8.09)	7.77 (7.08, 8.53)	8.05 (7.40, 8.81)	8.51 (7.71, 9.30)	9.33 (8.36, 10.15)	8.51 (7.23, 9.33)	9.02 (7.26, 10.12)	6.55 (5.01, 7.51)	14.85 (10.24, 17.55)
2021	0.19 (0.17, 0.22)	0.43 (0.37, 0.50)	0.90 (0.74, 1.06)	1.48 (1.25, 1.76)	2.37 (2.06, 2.73)	3.23 (2.83, 3.63)	4.32 (3.82, 4.84)	5.00 (4.43, 5.60)	6.32 (5.62, 7.09)	7.34 (6.61, 8.17)	7.85 (7.12, 8.70)	8.04 (7.36, 8.88)	8.55 (7.71, 9.42)	9.37 (8.40, 10.25)	8.49 (7.21, 9.35)	8.98 (7.24, 10.13)	6.49 (4.94, 7.47)	14.75 (10.10, 17.51)
2022	0.19 (0.17, 0.22)	0.44 (0.37, 0.52)	0.93 (0.75, 1.10)	1.52 (1.28, 1.82)	2.42 (2.10, 2.80)	3.29 (2.88, 3.72)	4.38 (3.86, 4.93)	5.05 (4.44, 5.70)	6.35 (5.60, 7.19)	7.34 (6.57, 8.25)	7.93 (7.15, 8.86)	8.03 (7.32, 8.94)	8.59 (7.71, 9.53)	9.41 (8.44, 10.34)	8.47 (7.20, 9.36)	8.94 (7.22, 10.14)	6.43 (4.88, 7.43)	14.65 (9.97, 17.47)
2023	0.20 (0.17, 0.22)	0.45 (0.38, 0.53)	0.96 (0.77, 1.14)	1.56 (1.31, 1.87)	2.46 (2.14, 2.87)	3.35 (2.92, 3.81)	4.43 (3.91, 5.02)	5.10 (4.45, 5.81)	6.37 (5.58, 7.29)	7.34 (6.52, 8.34)	8.01 (7.19, 9.03)	8.02 (7.27, 9.01)	8.63 (7.71, 9.64)	9.44 (8.48, 10.44)	8.45 (7.18, 9.37)	8.91 (7.21, 10.14)	6.36 (4.82, 7.39)	14.54 (9.83, 17.42)
2024	0.20 (0.18, 0.23)	0.46 (0.38, 0.54)	0.99 (0.79, 1.18)	1.60 (1.34, 1.93)	2.51 (2.17, 2.94)	3.41 (2.97, 3.90)	4.48 (3.96, 5.11)	5.15 (4.46, 5.91)	6.40 (5.55, 7.39)	7.34 (6.48, 8.42)	8.09 (7.22, 9.20)	8.01 (7.23, 9.08)	8.68 (7.71, 9.76)	9.48 (8.52, 10.53)	8.43 (7.16, 9.38)	8.87 (7.19, 10.15)	6.30 (4.75, 7.35)	14.44 (9.70, 17.38)
2025	0.20 (0.18, 0.23)	0.47 (0.39, 0.55)	1.02 (0.81, 1.22)	1.64 (1.37, 1.99)	2.55 (2.21, 3.01)	3.46 (3.02, 3.99)	4.54 (4.00, 5.20)	5.20 (4.47, 6.02)	6.42 (5.53, 7.49)	7.34 (6.44, 8.51)	8.17 (7.26, 9.37)	8.00 (7.19, 9.15)	8.72 (7.71, 9.87)	9.52 (8.55, 10.63)	8.40 (7.14, 9.39)	8.83 (7.17, 10.16)	6.24 (4.69, 7.31)	14.34 (9.56, 17.34)
2026	0.20 (0.18, 0.23)	0.48 (0.39, 0.57)	1.04 (0.83, 1.26)	1.68 (1.40, 2.05)	2.60 (2.25, 3.08)	3.52 (3.06, 4.08)	4.59 (4.05, 5.29)	5.25 (4.48, 6.12)	6.45 (5.51, 7.58)	7.33 (6.40, 8.59)	8.25 (7.29, 9.53)	8.00 (7.15, 9.22)	8.76 (7.71, 9.99)	9.55 (8.59, 10.72)	8.38 (7.12, 9.40)	8.79 (7.15, 10.16)	6.18 (4.62, 7.27)	14.24 (9.42, 17.29)
2027	0.21 (0.18, 0.24)	0.49 (0.40, 0.58)	1.07 (0.85, 1.30)	1.72 (1.43, 2.11)	2.64 (2.29, 3.15)	3.58 (3.11, 4.17)	4.64 (4.09, 5.39)	5.30 (4.49, 6.23)	6.48 (5.49, 7.68)	7.33 (6.36, 8.67)	8.33 (7.32, 9.70)	7.99 (7.11, 9.29)	8.80 (7.71, 10.10)	9.59 (8.63, 10.82)	8.36 (7.11, 9.41)	8.75 (7.13, 10.17)	6.12 (4.56, 7.23)	14.14 (9.29, 17.25)
2028	0.21 (0.18, 0.24)	0.50 (0.41, 0.59)	1.10 (0.87, 1.35)	1.76 (1.46, 2.16)	2.69 (2.33, 3.22)	3.64 (3.16, 4.26)	4.70 (4.14, 5.48)	5.35 (4.50, 6.33)	6.50 (5.47, 7.78)	7.33 (6.31, 8.76)	8.41 (7.36, 9.87)	7.98 (7.07, 9.36)	8.84 (7.71, 10.21)	9.63 (8.67, 10.92)	8.34 (7.09, 9.42)	8.72 (7.11, 10.18)	6.05 (4.50, 7.20)	14.04 (9.15, 17.21)
2029	0.21 (0.18, 0.24)	0.50 (0.41, 0.61)	1.13 (0.88, 1.39)	1.80 (1.49, 2.22)	2.73 (2.37, 3.29)	3.70 (3.21, 4.35)	4.75 (4.19, 5.57)	5.40 (4.51, 6.43)	6.53 (5.45, 7.88)	7.33 (6.27, 8.84)	8.49 (7.39, 10.04)	7.97 (7.03, 9.43)	8.88 (7.71, 10.33)	9.66 (8.71, 11.01)	8.32 (7.07, 9.43)	8.68 (7.10, 10.18)	5.99 (4.43, 7.16)	13.94 (9.02, 17.16)
2030	0.21 (0.18, 0.25)	0.51 (0.42, 0.62)	1.16 (0.90, 1.43)	1.84 (1.52, 2.28)	2.78 (2.41, 3.36)	3.76 (3.25, 4.44)	4.80 (4.23, 5.66)	5.45 (4.52, 6.54)	6.55 (5.42, 7.98)	7.33 (6.23, 8.93)	8.57 (7.43, 10.21)	7.96 (6.99, 9.49)	8.92 (7.71, 10.44)	9.70 (8.75, 11.11)	8.30 (7.05, 9.45)	8.64 (7.08, 10.19)	5.93 (4.37, 7.12)	13.83 (8.88, 17.12)

Table S3. The Death Rate of global Thyroid cancer burden Between 1990 and 2030, by ages.

death	10-14 years	15-19 years	20-24 years	25-29 years	30-34 years	35-39 years	40-44 years	45-49 years	50-54 years	55-59 years	60-64 years	65-69 years	70-74 years	75-79 years	80-84	85-89	90-94	95+ years
1990	0.02 (0.02, 0.02)	0.04 (0.03, 0.04)	0.07 (0.05, 0.08)	0.10 (0.08, 0.12)	0.12 (0.09, 0.14)	0.18 (0.15, 0.20)	0.27 (0.24, 0.31)	0.43 (0.39, 0.48)	0.77 (0.70, 0.84)	1.17 (1.08, 1.27)	1.68 (1.57, 1.86)	2.39 (2.26, 2.71)	3.31 (3.12, 3.78)	4.83 (4.53, 5.48)	6.12 (5.51, 6.86)	7.58 (6.60, 8.40)	8.18 (6.69, 9.17)	8.32 (6.44, 9.41)
1991	0.02 (0.02, 0.02)	0.04 (0.03, 0.04)	0.07 (0.05, 0.08)	0.10 (0.08, 0.12)	0.12 (0.10, 0.14)	0.17 (0.15, 0.20)	0.27 (0.24, 0.30)	0.43 (0.39, 0.47)	0.76 (0.70, 0.83)	1.17 (1.08, 1.26)	1.67 (1.57, 1.84)	2.38 (2.25, 2.68)	3.33 (3.13, 3.80)	4.80 (4.50, 5.45)	6.12 (5.53, 6.84)	7.53 (6.55, 8.34)	8.12 (6.63, 9.10)	8.32 (6.42, 9.42)
1992	0.02 (0.02, 0.02)	0.04 (0.03, 0.04)	0.07 (0.05, 0.08)	0.10 (0.08, 0.12)	0.12 (0.10, 0.14)	0.17 (0.15, 0.20)	0.27 (0.24, 0.30)	0.43 (0.39, 0.47)	0.76 (0.70, 0.83)	1.16 (1.08, 1.26)	1.67 (1.57, 1.84)	2.38 (2.25, 2.67)	3.35 (3.16, 3.84)	4.76 (4.46, 5.46)	6.14 (5.55, 6.88)	7.58 (6.60, 8.41)	8.22 (6.70, 9.18)	8.43 (6.48, 9.55)
1993	0.02 (0.02, 0.02)	0.04 (0.03, 0.04)	0.07 (0.05, 0.08)	0.10 (0.08, 0.12)	0.12 (0.10, 0.14)	0.18 (0.15, 0.20)	0.27 (0.24, 0.30)	0.43 (0.38, 0.46)	0.76 (0.70, 0.83)	1.17 (1.09, 1.27)	1.67 (1.57, 1.86)	2.39 (2.26, 2.68)	3.39 (3.19, 3.86)	4.75 (4.44, 5.46)	6.20 (5.59, 6.93)	7.68 (6.65, 8.53)	8.34 (6.79, 9.31)	8.61 (6.62, 9.74)
1994	0.02 (0.02, 0.03)	0.04 (0.03, 0.04)	0.07 (0.05, 0.08)	0.10 (0.08, 0.12)	0.12 (0.10, 0.14)	0.18 (0.15, 0.20)	0.28 (0.24, 0.31)	0.43 (0.39, 0.47)	0.76 (0.70, 0.83)	1.17 (1.09, 1.27)	1.66 (1.56, 1.83)	2.39 (2.26, 2.68)	3.39 (3.20, 3.85)	4.69 (4.37, 5.35)	6.19 (5.55, 6.89)	7.72 (6.68, 8.53)	8.37 (6.83, 9.36)	8.71 (6.71, 9.86)
1995	0.02 (0.02, 0.02)	0.04 (0.03, 0.04)	0.07 (0.06, 0.09)	0.10 (0.08, 0.12)	0.12 (0.10, 0.14)	0.18 (0.15, 0.20)	0.27 (0.24, 0.30)	0.43 (0.39, 0.47)	0.76 (0.69, 0.82)	1.16 (1.09, 1.25)	1.64 (1.54, 1.82)	2.38 (2.25, 2.68)	3.37 (3.19, 3.84)	4.67 (4.37, 5.33)	6.18 (5.52, 6.87)	7.76 (6.72, 8.56)	8.44 (6.85, 9.40)	8.81 (6.80, 10.00)
1996	0.02 (0.02, 0.02)	0.04 (0.03, 0.04)	0.07 (0.06, 0.09)	0.11 (0.08, 0.12)	0.13 (0.10, 0.14)	0.18 (0.15, 0.20)	0.27 (0.24, 0.30)	0.43 (0.39, 0.47)	0.76 (0.69, 0.82)	1.14 (1.07, 1.23)	1.63 (1.53, 1.77)	2.36 (2.24, 2.63)	3.33 (3.17, 3.77)	4.67 (4.38, 5.28)	6.14 (5.50, 6.80)	7.78 (6.71, 8.57)	8.45 (6.87, 9.42)	8.89 (6.81, 10.07)
1997	0.02 (0.02, 0.02)	0.04 (0.03, 0.04)	0.07 (0.06, 0.09)	0.11 (0.08, 0.13)	0.13 (0.10, 0.15)	0.18 (0.15, 0.20)	0.27 (0.24, 0.30)	0.43 (0.38, 0.47)	0.75 (0.68, 0.81)	1.13 (1.06, 1.21)	1.62 (1.53, 1.76)	2.35 (2.23, 2.61)	3.31 (3.14, 3.71)	4.69 (4.41, 5.26)	6.09 (5.46, 6.75)	7.79 (6.73, 8.52)	8.50 (6.90, 9.45)	8.98 (6.87, 10.15)
1998	0.02 (0.02, 0.02)	0.04 (0.03, 0.04)	0.08 (0.06, 0.09)	0.11 (0.08, 0.13)	0.13 (0.10, 0.15)	0.18 (0.15, 0.20)	0.27 (0.23, 0.30)	0.43 (0.38, 0.46)	0.73 (0.67, 0.79)	1.12 (1.04, 1.20)	1.62 (1.52, 1.74)	2.35 (2.22, 2.59)	3.30 (3.13, 3.68)	4.73 (4.42, 5.24)	6.06 (5.42, 6.67)	7.81 (6.76, 8.52)	8.52 (6.91, 9.47)	9.00 (6.86, 10.17)
1999	0.02 (0.02, 0.03)	0.04 (0.03, 0.04)	0.08 (0.06, 0.10)	0.11 (0.09, 0.13)	0.13 (0.10, 0.15)	0.18 (0.15, 0.20)	0.27 (0.23, 0.30)	0.43 (0.38, 0.46)	0.73 (0.67, 0.78)	1.12 (1.04, 1.20)	1.61 (1.51, 1.72)	2.33 (2.20, 2.55)	3.30 (3.13, 3.66)	4.76 (4.44, 5.28)	6.00 (5.37, 6.61)	7.84 (6.76, 8.55)	8.63 (6.98, 9.60)	9.11 (6.96, 10.30)
2000	0.02 (0.02, 0.03)	0.04 (0.03, 0.04)	0.08 (0.06, 0.10)	0.11 (0.09, 0.14)	0.13 (0.10, 0.15)	0.18 (0.15, 0.20)	0.27 (0.23, 0.29)	0.43 (0.38, 0.46)	0.73 (0.66, 0.77)	1.12 (1.03, 1.20)	1.60 (1.50, 1.72)	2.33 (2.20, 2.54)	3.31 (3.13, 3.63)	4.75 (4.43, 5.21)	5.96 (5.29, 6.55)	7.80 (6.70, 8.48)	8.58 (6.91, 9.53)	9.14 (6.95, 10.33)
2001	0.02 (0.02, 0.02)	0.04 (0.03, 0.04)	0.08 (0.06, 0.09)	0.11 (0.09, 0.13)	0.13 (0.10, 0.14)	0.18 (0.15, 0.20)	0.27 (0.23, 0.29)	0.42 (0.38, 0.45)	0.73 (0.66, 0.78)	1.12 (1.02, 1.19)	1.59 (1.50, 1.69)	2.33 (2.18, 2.51)	3.32 (3.14, 3.63)	4.75 (4.43, 5.17)	5.95 (5.28, 6.46)	7.76 (6.66, 8.43)	8.62 (6.92, 9.58)	9.22 (6.99, 10.42)
2002	0.02 (0.02, 0.02)	0.04 (0.03, 0.04)	0.08 (0.06, 0.09)	0.11 (0.09, 0.13)	0.12 (0.10, 0.14)	0.18 (0.15, 0.20)	0.26 (0.23, 0.29)	0.42 (0.37, 0.45)	0.73 (0.67, 0.78)	1.12 (1.03, 1.19)	1.60 (1.50, 1.71)	2.33 (2.19, 2.52)	3.33 (3.13, 3.63)	4.79 (4.47, 5.22)	6.00 (5.30, 6.51)	7.75 (6.63, 8.44)	8.74 (7.01, 9.70)	9.50 (7.17, 10.76)
2003	0.02 (0.02, 0.02)	0.04 (0.03, 0.04)	0.07 (0.06, 0.09)	0.11 (0.09, 0.12)	0.12 (0.10, 0.13)	0.17 (0.15, 0.19)	0.26 (0.23, 0.28)	0.41 (0.37, 0.44)	0.73 (0.67, 0.78)	1.12 (1.03, 1.19)	1.61 (1.51, 1.71)	2.34 (2.18, 2.50)	3.33 (3.14, 3.60)	4.81 (4.46, 5.19)	6.05 (5.33, 6.48)	7.71 (6.57, 8.43)	8.76 (7.01, 9.73)	9.74 (7.33, 11.02)

2004	0.02 (0.02, 0.02)	0.03 (0.03, 0.04)	0.07 (0.06, 0.08)	0.11 (0.09, 0.12)	0.12 (0.10, 0.13)	0.17 (0.15, 0.19)	0.26 (0.23, 0.28)	0.41 (0.37, 0.44)	0.72 (0.66, 0.76)	1.11 (1.01, 1.17)	1.59 (1.48, 1.68)	2.32 (2.15, 2.48)	3.29 (3.09, 3.56)	4.80 (4.45, 5.15)	6.07 (5.34, 6.51)	7.62 (6.47, 8.33)	8.79 (6.99, 9.77)	10.09 (7.57, 11.40)
2005	0.02 (0.02, 0.02)	0.04 (0.03, 0.04)	0.07 (0.06, 0.08)	0.11 (0.09, 0.12)	0.12 (0.10, 0.13)	0.17 (0.15, 0.19)	0.26 (0.23, 0.28)	0.42 (0.38, 0.44)	0.72 (0.66, 0.76)	1.11 (1.03, 1.17)	1.59 (1.47, 1.68)	2.33 (2.15, 2.47)	3.31 (3.10, 3.54)	4.85 (4.49, 5.16)	6.12 (5.39, 6.53)	7.67 (6.49, 8.41)	8.88 (7.05, 9.91)	10.49 (7.83, 11.86)
2006	0.02 (0.02, 0.02)	0.03 (0.03, 0.04)	0.07 (0.06, 0.08)	0.11 (0.09, 0.12)	0.12 (0.10, 0.13)	0.17 (0.15, 0.19)	0.26 (0.23, 0.28)	0.41 (0.37, 0.44)	0.71 (0.65, 0.76)	1.11 (1.02, 1.17)	1.58 (1.46, 1.67)	2.32 (2.14, 2.44)	3.32 (3.09, 3.54)	4.88 (4.50, 5.14)	6.13 (5.38, 6.50)	7.71 (6.49, 8.39)	8.94 (7.05, 9.99)	10.69 (7.94, 12.13)
2007	0.02 (0.02, 0.02)	0.03 (0.03, 0.04)	0.07 (0.06, 0.08)	0.11 (0.09, 0.12)	0.12 (0.10, 0.13)	0.17 (0.15, 0.19)	0.26 (0.23, 0.28)	0.41 (0.37, 0.44)	0.71 (0.64, 0.75)	1.11 (1.01, 1.16)	1.58 (1.44, 1.66)	2.32 (2.12, 2.44)	3.31 (3.06, 3.50)	4.89 (4.49, 5.13)	6.13 (5.35, 6.50)	7.77 (6.50, 8.45)	8.97 (7.09, 10.02)	10.87 (8.01, 12.37)
2008	0.02 (0.02, 0.02)	0.03 (0.03, 0.04)	0.07 (0.06, 0.08)	0.11 (0.09, 0.12)	0.12 (0.10, 0.13)	0.17 (0.15, 0.19)	0.26 (0.23, 0.28)	0.42 (0.37, 0.44)	0.72 (0.65, 0.75)	1.11 (1.00, 1.16)	1.57 (1.44, 1.65)	2.32 (2.10, 2.43)	3.32 (3.05, 3.50)	4.94 (4.51, 5.19)	6.21 (5.40, 6.60)	7.89 (6.59, 8.61)	9.07 (7.11, 10.14)	10.97 (8.07, 12.51)
2009	0.02 (0.02, 0.02)	0.03 (0.03, 0.04)	0.07 (0.06, 0.08)	0.11 (0.09, 0.12)	0.12 (0.10, 0.13)	0.17 (0.15, 0.19)	0.26 (0.22, 0.27)	0.42 (0.37, 0.44)	0.72 (0.64, 0.76)	1.10 (0.99, 1.15)	1.55 (1.42, 1.64)	2.32 (2.10, 2.43)	3.30 (3.04, 3.47)	4.94 (4.48, 5.19)	6.21 (5.37, 6.60)	7.94 (6.58, 8.66)	9.04 (7.06, 10.10)	10.95 (8.00, 12.50)
2010	0.02 (0.02, 0.02)	0.03 (0.03, 0.04)	0.07 (0.06, 0.08)	0.11 (0.09, 0.12)	0.12 (0.10, 0.13)	0.17 (0.15, 0.19)	0.26 (0.22, 0.27)	0.42 (0.37, 0.45)	0.72 (0.64, 0.76)	1.10 (0.99, 1.16)	1.55 (1.42, 1.63)	2.33 (2.10, 2.44)	3.30 (3.03, 3.47)	4.95 (4.47, 5.20)	6.25 (5.39, 6.66)	7.97 (6.60, 8.68)	9.16 (7.17, 10.26)	11.11 (8.10, 12.73)
2011	0.02 (0.02, 0.02)	0.03 (0.03, 0.04)	0.07 (0.06, 0.08)	0.11 (0.09, 0.12)	0.12 (0.11, 0.13)	0.17 (0.15, 0.18)	0.25 (0.22, 0.27)	0.41 (0.37, 0.44)	0.72 (0.64, 0.76)	1.10 (0.99, 1.16)	1.54 (1.41, 1.61)	2.30 (2.10, 2.42)	3.27 (3.01, 3.44)	4.94 (4.42, 5.22)	6.25 (5.36, 6.66)	8.00 (6.58, 8.72)	9.27 (7.25, 10.41)	11.35 (8.25, 13.00)
2012	0.02 (0.02, 0.02)	0.03 (0.03, 0.04)	0.07 (0.06, 0.08)	0.10 (0.09, 0.11)	0.12 (0.10, 0.13)	0.17 (0.15, 0.18)	0.25 (0.22, 0.27)	0.41 (0.37, 0.44)	0.71 (0.64, 0.75)	1.10 (0.99, 1.16)	1.53 (1.40, 1.61)	2.27 (2.06, 2.38)	3.25 (2.96, 3.43)	4.92 (4.39, 5.18)	6.23 (5.31, 6.66)	8.01 (6.60, 8.72)	9.37 (7.37, 10.52)	11.46 (8.32, 13.16)
2013	0.02 (0.02, 0.02)	0.03 (0.03, 0.04)	0.07 (0.06, 0.08)	0.10 (0.09, 0.11)	0.12 (0.10, 0.13)	0.17 (0.15, 0.18)	0.25 (0.22, 0.27)	0.41 (0.36, 0.43)	0.71 (0.63, 0.75)	1.09 (0.98, 1.15)	1.51 (1.39, 1.59)	2.22 (2.02, 2.32)	3.21 (2.93, 3.38)	4.89 (4.33, 5.15)	6.26 (5.31, 6.67)	8.04 (6.61, 8.75)	9.45 (7.40, 10.61)	11.61 (8.41, 13.33)
2014	0.02 (0.02, 0.02)	0.03 (0.03, 0.04)	0.07 (0.06, 0.08)	0.10 (0.08, 0.11)	0.11 (0.10, 0.13)	0.17 (0.15, 0.18)	0.25 (0.22, 0.27)	0.40 (0.35, 0.42)	0.71 (0.63, 0.75)	1.09 (0.98, 1.15)	1.50 (1.38, 1.58)	2.16 (1.98, 2.27)	3.13 (2.88, 3.31)	4.84 (4.31, 5.09)	6.26 (5.32, 6.71)	7.98 (6.56, 8.70)	9.43 (7.38, 10.58)	11.71 (8.44, 13.45)
2015	0.02 (0.02, 0.02)	0.03 (0.03, 0.04)	0.07 (0.06, 0.08)	0.09 (0.08, 0.11)	0.11 (0.10, 0.13)	0.17 (0.15, 0.18)	0.25 (0.22, 0.27)	0.39 (0.35, 0.42)	0.70 (0.63, 0.76)	1.10 (1.00, 1.17)	1.50 (1.38, 1.58)	2.16 (1.97, 2.26)	3.12 (2.88, 3.30)	4.80 (4.31, 5.05)	6.19 (5.26, 6.62)	7.92 (6.53, 8.64)	9.40 (7.34, 10.58)	11.83 (8.48, 13.62)
2016	0.02 (0.02, 0.02)	0.03 (0.03, 0.04)	0.07 (0.06, 0.08)	0.09 (0.08, 0.11)	0.11 (0.10, 0.12)	0.17 (0.15, 0.18)	0.25 (0.22, 0.27)	0.39 (0.35, 0.42)	0.70 (0.62, 0.75)	1.10 (0.99, 1.17)	1.50 (1.38, 1.59)	2.16 (1.98, 2.27)	3.12 (2.87, 3.29)	4.74 (4.27, 5.02)	6.12 (5.23, 6.55)	7.85 (6.45, 8.58)	9.36 (7.26, 10.55)	11.88 (8.46, 13.68)
2017	0.02 (0.02, 0.02)	0.03 (0.03, 0.04)	0.07 (0.06, 0.08)	0.10 (0.08, 0.11)	0.11 (0.10, 0.13)	0.17 (0.15, 0.18)	0.25 (0.22, 0.27)	0.39 (0.34, 0.42)	0.69 (0.61, 0.74)	1.09 (0.97, 1.17)	1.49 (1.36, 1.59)	2.14 (1.96, 2.27)	3.09 (2.84, 3.29)	4.69 (4.21, 4.97)	6.07 (5.20, 6.53)	7.76 (6.36, 8.47)	9.19 (7.10, 10.40)	11.67 (8.30, 13.48)
2018	0.02 (0.02, 0.02)	0.03 (0.03, 0.04)	0.07 (0.06, 0.08)	0.10 (0.08, 0.11)	0.11 (0.10, 0.13)	0.17 (0.15, 0.19)	0.25 (0.22, 0.27)	0.39 (0.34, 0.42)	0.68 (0.61, 0.75)	1.08 (0.96, 1.16)	1.49 (1.37, 1.60)	2.13 (1.95, 2.28)	3.08 (2.84, 3.30)	4.69 (4.21, 5.00)	6.08 (5.20, 6.56)	7.76 (6.38, 8.53)	9.16 (7.07, 10.36)	11.61 (8.29, 13.45)
2019	0.02 (0.02, 0.02)	0.03 (0.03, 0.04)	0.07 (0.06, 0.09)	0.10 (0.09, 0.12)	0.11 (0.10, 0.13)	0.17 (0.15, 0.19)	0.25 (0.22, 0.28)	0.39 (0.34, 0.42)	0.68 (0.60, 0.74)	1.06 (0.96, 1.16)	1.50 (1.37, 1.62)	2.14 (1.96, 2.30)	3.07 (2.82, 3.29)	4.69 (4.19, 5.03)	6.07 (5.21, 6.61)	7.79 (6.37, 8.60)	9.15 (6.99, 10.36)	11.57 (8.24, 13.42)

2020	0.02 (0.02, 0.02)	0.03 (0.03, 0.04)	0.07 (0.06, 0.09)	0.10 (0.09, 0.12)	0.11 (0.10, 0.13)	0.17 (0.15, 0.19)	0.25 (0.22, 0.28)	0.38 (0.34, 0.42)	0.67 (0.60, 0.74)	1.06 (0.95, 1.15)	1.50 (1.37, 1.62)	2.13 (1.95, 2.30)	3.05 (2.80, 3.30)	4.65 (4.16, 5.01)	6.03 (5.19, 6.58)	7.73 (6.32, 8.55)	9.05 (6.89, 10.28)	11.49 (8.17, 13.36)
2021	0.02 (0.01, 0.02)	0.03 (0.03, 0.04)	0.08 (0.06, 0.09)	0.10 (0.09, 0.12)	0.11 (0.10, 0.13)	0.17 (0.15, 0.19)	0.25 (0.22, 0.28)	0.38 (0.34, 0.42)	0.67 (0.59, 0.74)	1.04 (0.94, 1.15)	1.51 (1.37, 1.63)	2.13 (1.94, 2.31)	3.04 (2.79, 3.30)	4.63 (4.14, 5.01)	6.00 (5.18, 6.59)	7.71 (6.29, 8.55)	8.99 (6.80, 10.22)	11.41 (8.11, 13.29)
2022	0.02 (0.01, 0.02)	0.03 (0.03, 0.04)	0.08 (0.06, 0.09)	0.11 (0.09, 0.12)	0.12 (0.10, 0.13)	0.17 (0.15, 0.20)	0.25 (0.22, 0.28)	0.38 (0.34, 0.42)	0.66 (0.59, 0.74)	1.03 (0.93, 1.15)	1.51 (1.37, 1.64)	2.13 (1.94, 2.33)	3.03 (2.78, 3.30)	4.61 (4.11, 5.02)	5.98 (5.17, 6.60)	7.68 (6.27, 8.56)	8.92 (6.71, 10.16)	11.32 (8.04, 13.22)
2023	0.02 (0.01, 0.02)	0.03 (0.03, 0.04)	0.08 (0.06, 0.10)	0.11 (0.09, 0.13)	0.12 (0.10, 0.13)	0.18 (0.15, 0.20)	0.26 (0.22, 0.28)	0.38 (0.33, 0.42)	0.66 (0.58, 0.74)	1.02 (0.93, 1.14)	1.51 (1.37, 1.65)	2.12 (1.94, 2.34)	3.01 (2.76, 3.30)	4.59 (4.09, 5.02)	5.96 (5.17, 6.61)	7.66 (6.24, 8.56)	8.85 (6.62, 10.10)	11.24 (7.98, 13.15)
2024	0.02 (0.01, 0.02)	0.03 (0.03, 0.04)	0.08 (0.07, 0.10)	0.11 (0.09, 0.13)	0.12 (0.10, 0.13)	0.18 (0.15, 0.20)	0.26 (0.23, 0.29)	0.38 (0.33, 0.42)	0.65 (0.57, 0.74)	1.01 (0.92, 1.14)	1.51 (1.37, 1.66)	2.12 (1.93, 2.35)	3.00 (2.75, 3.31)	4.57 (4.06, 5.02)	5.94 (5.16, 6.62)	7.64 (6.21, 8.57)	8.79 (6.53, 10.04)	11.15 (7.91, 13.08)
2025	0.02 (0.01, 0.02)	0.03 (0.03, 0.04)	0.08 (0.07, 0.10)	0.11 (0.10, 0.13)	0.12 (0.10, 0.13)	0.18 (0.16, 0.20)	0.26 (0.23, 0.29)	0.38 (0.33, 0.42)	0.65 (0.57, 0.73)	1.00 (0.91, 1.13)	1.52 (1.37, 1.67)	2.12 (1.93, 2.36)	2.99 (2.73, 3.31)	4.55 (4.04, 5.03)	5.91 (5.15, 6.63)	7.61 (6.18, 8.58)	8.72 (6.44, 9.98)	11.07 (7.85, 13.01)
2026	0.02 (0.01, 0.02)	0.03 (0.03, 0.04)	0.08 (0.07, 0.10)	0.11 (0.10, 0.14)	0.12 (0.10, 0.13)	0.18 (0.16, 0.20)	0.26 (0.23, 0.29)	0.38 (0.33, 0.43)	0.64 (0.56, 0.73)	0.99 (0.90, 1.13)	1.52 (1.37, 1.68)	2.12 (1.93, 2.38)	2.97 (2.72, 3.31)	4.53 (4.02, 5.03)	5.89 (5.14, 6.63)	7.59 (6.16, 8.58)	8.65 (6.35, 9.92)	10.98 (7.78, 12.94)
2027	0.02 (0.01, 0.02)	0.03 (0.03, 0.04)	0.09 (0.07, 0.11)	0.12 (0.10, 0.14)	0.12 (0.10, 0.14)	0.18 (0.16, 0.21)	0.26 (0.23, 0.29)	0.37 (0.32, 0.43)	0.64 (0.56, 0.73)	0.98 (0.89, 1.12)	1.52 (1.37, 1.69)	2.12 (1.92, 2.39)	2.96 (2.71, 3.32)	4.51 (3.99, 5.04)	5.87 (5.14, 6.64)	7.57 (6.13, 8.59)	8.59 (6.26, 9.86)	10.90 (7.72, 12.87)
2028	0.02 (0.01, 0.02)	0.03 (0.03, 0.04)	0.09 (0.07, 0.11)	0.12 (0.10, 0.14)	0.12 (0.10, 0.14)	0.18 (0.16, 0.21)	0.26 (0.23, 0.30)	0.37 (0.32, 0.43)	0.63 (0.55, 0.73)	0.97 (0.88, 1.12)	1.53 (1.37, 1.70)	2.11 (1.92, 2.40)	2.95 (2.69, 3.32)	4.49 (3.97, 5.04)	5.84 (5.13, 6.65)	7.54 (6.10, 8.59)	8.52 (6.17, 9.80)	10.81 (7.65, 12.80)
2029	0.02 (0.01, 0.02)	0.03 (0.03, 0.04)	0.09 (0.07, 0.11)	0.12 (0.10, 0.14)	0.12 (0.11, 0.14)	0.18 (0.16, 0.21)	0.26 (0.23, 0.30)	0.37 (0.32, 0.43)	0.63 (0.54, 0.73)	0.95 (0.88, 1.11)	1.53 (1.38, 1.71)	2.11 (1.92, 2.41)	2.94 (2.68, 3.32)	4.47 (3.95, 5.05)	5.82 (5.12, 6.66)	7.52 (6.08, 8.60)	8.45 (6.08, 9.74)	10.73 (7.59, 12.72)
2030	0.02 (0.01, 0.02)	0.03 (0.03, 0.04)	0.09 (0.07, 0.12)	0.12 (0.10, 0.15)	0.12 (0.11, 0.14)	0.18 (0.16, 0.21)	0.26 (0.23, 0.30)	0.37 (0.32, 0.43)	0.62 (0.54, 0.73)	0.94 (0.87, 1.11)	1.53 (1.38, 1.72)	2.11 (1.91, 2.43)	2.92 (2.66, 3.32)	4.45 (3.92, 5.05)	5.80 (5.11, 6.67)	7.50 (6.05, 8.60)	8.38 (5.99, 9.68)	10.64 (7.52, 12.65)

Table S4. The DALY Rate of global Thyroid cancer burden Between 1990 and 2030, by ages.

daly	10-14 years	15-19 years	20-24 years	25-29 years	30-34 years	35-39 years	40-44 years	45-49 years	50-54 years	55-59 years	60-64 years	65-69 years	70-74 years	75-79 years	80-84	85-89	90-94	95+ years
1990	1.67 (1.39, 1.96)	2.66 (2.19, 3.18)	4.75 (3.68, 5.78)	6.47 (5.04, 7.76)	7.30 (5.92, 8.49)	10.02 (8.49, 11.59)	14.01 (12.30, 15.71)	19.69 (17.67, 21.71)	30.76 (27.90, 33.75)	40.49 (37.43, 44.36)	49.43 (46.39, 54.31)	58.78 (55.49, 66.21)	66.24 (62.15, 75.62)	76.01 (71.28, 86.09)	73.56 (66.29, 82.51)	69.54 (60.46, 77.19)	57.53 (47.20, 64.52)	45.49 (35.34, 51.52)
1991	1.68 (1.40, 1.98)	2.70 (2.23, 3.21)	4.79 (3.75, 5.77)	6.44 (5.12, 7.72)	7.35 (6.07, 8.57)	9.98 (8.44, 11.43)	13.99 (12.28, 15.70)	19.63 (17.83, 21.67)	30.59 (28.01, 33.33)	40.34 (37.50, 44.02)	49.26 (46.21, 54.23)	58.61 (55.29, 66.16)	66.53 (62.51, 76.08)	75.58 (70.85, 85.65)	73.52 (66.21, 82.35)	69.07 (60.10, 76.71)	57.06 (46.72, 64.05)	45.46 (35.25, 51.50)
19	1.68 (1.41,	2.74 (2.26,	4.86 (3.76,	6.46 (5.07,	7.42 (6.10,	10.03 (8.52,	14.11 (12.38,	19.59 (17.51,	30.63 (27.95,	40.29 (37.13,	49.29 (46.04,	58.62 (55.23,	67.10 (63.17,	75.10 (70.33,	73.89 (66.65,	69.51 (60.58,	57.76 (47.15,	46.01 (35.56,

92	1.99)	3.25)	5.82)	7.82)	8.56)	11.46)	15.73)	21.60)	33.52)	43.85)	54.35)	66.08)	76.99)	85.57)	82.53)	77.20)	64.54)	52.20)
1993	1.68 (1.40, 1.98)	2.76 (2.30, 3.28)	4.88 (3.80, 5.82)	6.46 (5.11, 7.72)	7.44 (6.08, 8.63)	10.12 (8.65, 11.54)	14.25 (12.49, 15.85)	19.68 (17.82, 21.56)	30.76 (28.04, 33.46)	40.59 (37.65, 44.08)	49.42 (46.21, 54.66)	58.93 (55.60, 66.38)	67.92 (63.97, 77.78)	74.91 (70.17, 85.57)	74.57 (67.17, 83.46)	70.50 (61.08, 78.20)	58.59 (47.87, 65.53)	47.01 (36.28, 53.29)
1994	1.70 (1.44, 1.99)	2.74 (2.30, 3.22)	4.97 (3.89, 5.89)	6.74 (5.33, 7.98)	7.70 (6.27, 8.84)	10.25 (8.81, 11.67)	14.39 (12.64, 15.97)	20.02 (18.09, 21.85)	30.84 (28.18, 33.62)	40.58 (37.93, 44.20)	49.19 (45.97, 53.95)	58.83 (55.48, 65.99)	68.06 (64.20, 77.50)	73.97 (69.01, 84.27)	74.49 (66.92, 82.60)	70.84 (61.23, 78.43)	58.82 (48.18, 65.71)	47.49 (36.84, 54.08)
1995	1.72 (1.45, 1.98)	2.73 (2.31, 3.19)	5.07 (3.94, 6.01)	6.95 (5.45, 8.18)	7.87 (6.38, 9.02)	10.29 (8.86, 11.69)	14.33 (12.50, 15.92)	20.14 (18.17, 21.98)	30.60 (27.81, 33.22)	40.23 (37.54, 43.75)	48.59 (45.42, 53.35)	58.65 (55.49, 65.77)	67.72 (63.91, 77.22)	73.76 (68.92, 84.27)	74.36 (66.54, 82.14)	71.27 (61.62, 78.48)	59.30 (48.33, 66.13)	48.05 (37.19, 54.80)
1996	1.72 (1.45, 1.99)	2.73 (2.32, 3.17)	5.16 (4.06, 6.12)	7.03 (5.51, 8.27)	7.91 (6.45, 9.04)	10.21 (8.77, 11.50)	14.19 (12.50, 15.67)	20.12 (18.14, 21.83)	30.55 (27.77, 33.17)	39.89 (37.07, 42.93)	48.16 (45.03, 52.60)	58.30 (55.09, 64.51)	67.05 (63.43, 75.92)	73.77 (69.11, 83.29)	73.96 (66.21, 81.82)	71.44 (61.75, 78.53)	59.36 (48.45, 66.07)	48.43 (37.32, 55.03)
1997	1.72 (1.45, 1.96)	2.75 (2.32, 3.21)	5.30 (4.11, 6.32)	7.16 (5.54, 8.41)	8.00 (6.49, 9.19)	10.26 (8.82, 11.65)	14.17 (12.35, 15.67)	20.04 (17.88, 21.75)	30.23 (27.32, 32.84)	39.58 (36.88, 42.58)	48.07 (45.03, 52.05)	58.07 (54.89, 64.55)	66.58 (63.10, 74.16)	74.14 (69.49, 83.07)	73.38 (65.63, 81.01)	71.53 (61.80, 78.41)	59.72 (48.54, 66.39)	48.88 (37.49, 55.33)
1998	1.73 (1.47, 1.99)	2.77 (2.35, 3.24)	5.44 (4.18, 6.57)	7.35 (5.66, 8.66)	8.13 (6.55, 9.31)	10.32 (8.87, 11.63)	14.18 (12.32, 15.69)	19.91 (17.84, 21.64)	29.83 (27.06, 32.19)	39.25 (36.40, 42.11)	47.94 (44.85, 51.86)	57.96 (54.61, 63.67)	66.41 (62.91, 74.03)	74.80 (69.91, 83.01)	72.97 (65.07, 80.55)	71.77 (62.18, 78.28)	59.83 (48.51, 66.48)	48.96 (37.52, 55.45)
1999	1.75 (1.48, 2.01)	2.82 (2.37, 3.27)	5.57 (4.29, 6.73)	7.52 (5.77, 8.89)	8.19 (6.57, 9.38)	10.40 (8.92, 11.70)	14.22 (12.33, 15.72)	20.01 (17.84, 21.84)	29.67 (27.01, 32.04)	39.34 (36.27, 42.19)	47.73 (44.75, 51.13)	57.62 (54.21, 63.14)	66.45 (62.85, 73.66)	75.32 (70.43, 83.48)	72.26 (64.28, 79.63)	72.07 (62.48, 78.59)	60.61 (48.97, 67.38)	49.56 (37.88, 56.08)
2000	1.77 (1.49, 2.03)	2.84 (2.38, 3.30)	5.62 (4.30, 6.76)	7.62 (5.85, 9.01)	8.25 (6.52, 9.41)	10.45 (9.00, 11.78)	14.22 (12.34, 15.65)	20.03 (17.82, 21.66)	29.72 (27.06, 31.94)	39.38 (36.00, 42.10)	47.58 (44.34, 50.69)	57.55 (54.15, 62.71)	66.60 (63.04, 73.10)	75.24 (70.46, 82.42)	71.78 (63.97, 78.55)	71.67 (62.04, 77.97)	60.30 (48.46, 66.85)	49.63 (37.78, 56.28)
2001	1.71 (1.47, 1.96)	2.75 (2.33, 3.19)	5.52 (4.27, 6.59)	7.58 (5.91, 8.92)	8.15 (6.64, 9.26)	10.38 (8.89, 11.71)	14.11 (12.36, 15.54)	19.80 (17.66, 21.44)	29.72 (27.01, 32.03)	39.17 (35.97, 41.78)	47.43 (44.20, 50.45)	57.52 (53.92, 62.40)	66.82 (63.11, 72.94)	75.29 (70.43, 81.73)	71.67 (63.63, 78.13)	71.34 (61.35, 77.82)	60.55 (48.68, 67.31)	50.01 (37.87, 56.73)
2002	1.61 (1.40, 1.83)	2.69 (2.31, 3.09)	5.41 (4.25, 6.45)	7.34 (5.81, 8.60)	7.79 (6.38, 8.81)	10.34 (8.96, 11.71)	14.12 (12.39, 15.57)	19.67 (17.69, 21.32)	29.82 (27.15, 32.09)	39.39 (36.03, 42.17)	47.84 (44.50, 50.99)	57.80 (54.13, 62.81)	67.17 (63.29, 73.30)	75.87 (70.60, 82.37)	72.33 (64.01, 78.83)	71.29 (60.94, 77.92)	61.40 (49.23, 68.22)	51.52 (38.91, 58.44)
2003	1.54 (1.35, 1.74)	2.68 (2.30, 3.06)	5.25 (4.29, 6.07)	7.13 (5.80, 8.22)	7.63 (6.34, 8.54)	10.13 (8.87, 11.30)	14.04 (12.38, 15.40)	19.60 (17.62, 21.25)	29.85 (27.27, 32.04)	39.48 (36.26, 42.05)	48.13 (44.89, 51.05)	58.06 (54.16, 62.44)	67.18 (63.47, 72.46)	76.20 (70.78, 82.26)	72.96 (64.35, 78.57)	70.92 (60.51, 77.53)	61.47 (49.23, 68.38)	52.81 (39.84, 59.89)
2004	1.53 (1.35, 1.73)	2.68 (2.31, 3.05)	5.23 (4.26, 6.01)	7.10 (5.80, 8.11)	7.66 (6.49, 8.50)	10.02 (8.81, 11.06)	13.90 (12.30, 15.19)	19.69 (17.72, 21.23)	29.52 (26.85, 31.59)	39.14 (35.64, 41.69)	47.72 (43.82, 50.53)	57.57 (53.28, 61.37)	66.49 (62.10, 71.94)	76.02 (70.34, 81.71)	73.30 (64.48, 78.54)	70.13 (59.67, 76.51)	61.63 (49.08, 68.70)	54.65 (41.05, 61.90)
2005	1.56 (1.37, 1.77)	2.70 (2.32, 3.06)	5.27 (4.32, 6.06)	7.18 (5.92, 8.20)	7.63 (6.47, 8.48)	10.12 (8.93, 11.22)	13.87 (12.32, 15.15)	19.90 (17.78, 21.46)	29.63 (27.04, 31.62)	39.43 (35.97, 41.84)	47.74 (43.94, 50.49)	57.90 (53.44, 61.53)	66.91 (62.55, 71.67)	76.90 (71.00, 81.76)	73.87 (65.13, 78.90)	70.53 (59.85, 77.41)	62.23 (49.55, 69.51)	56.86 (42.53, 64.42)
2006	1.56 (1.36, 1.78)	2.67 (2.32, 2.99)	5.26 (4.31, 6.05)	7.19 (5.92, 8.20)	7.68 (6.49, 8.52)	10.23 (8.89, 11.29)	13.93 (12.38, 15.29)	19.80 (17.80, 21.36)	29.42 (26.78, 31.52)	39.40 (35.90, 41.94)	47.51 (43.42, 50.44)	57.84 (53.20, 60.96)	67.08 (62.29, 71.29)	77.40 (71.35, 81.38)	74.09 (65.13, 78.86)	70.89 (59.82, 77.44)	62.67 (49.66, 70.03)	57.92 (43.11, 65.76)
2007	1.55 (1.35, 1.76)	2.64 (2.30, 2.96)	5.23 (4.30, 6.06)	7.15 (5.91, 8.14)	7.75 (6.56, 8.60)	10.28 (8.97, 11.24)	14.06 (12.42, 15.36)	19.76 (17.75, 21.32)	29.42 (26.59, 31.46)	39.39 (36.02, 41.75)	47.48 (43.19, 50.25)	57.77 (53.10, 60.90)	66.91 (61.90, 70.78)	77.65 (71.22, 81.62)	74.14 (64.75, 78.67)	71.47 (60.06, 77.98)	62.88 (49.77, 70.24)	58.89 (43.50, 67.17)
20	1.55 (1.36,	2.65 (2.32,	5.21 (4.33,	7.19 (5.99,	7.89 (6.71,	10.39 (9.01,	14.21 (12.43,	19.93 (17.68,	29.71 (26.70,	39.45 (36.04,	47.47 (42.98,	57.90 (52.98,	67.13 (61.43,	78.48 (71.43,	75.06 (65.16,	72.66 (60.55,	63.58 (49.96,	59.43 (43.88,

08	1.77)	2.96)	5.97)	8.09)	8.77)	11.33)	15.48)	21.51)	31.73)	41.70)	50.03)	61.07)	70.97)	82.62)	79.95)	79.35)	71.10)	67.83)
2009	1.58 (1.39, 1.79)	2.65 (2.32, 2.96)	5.14 (4.29, 5.89)	7.26 (6.06, 8.16)	7.94 (6.68, 8.81)	10.39 (8.98, 11.32)	14.02 (12.18, 15.25)	20.07 (17.76, 21.68)	29.76 (26.58, 31.67)	39.19 (35.31, 41.46)	47.00 (42.78, 49.67)	58.01 (52.80, 61.20)	66.96 (61.24, 70.49)	78.52 (71.13, 82.58)	75.14 (64.85, 80.04)	73.09 (60.84, 79.75)	63.39 (49.58, 70.85)	59.25 (43.35, 67.89)
2010	1.60 (1.39, 1.80)	2.67 (2.34, 2.99)	5.17 (4.33, 5.91)	7.40 (6.16, 8.29)	8.02 (6.82, 8.88)	10.46 (9.05, 11.40)	14.00 (12.08, 15.20)	20.13 (17.81, 21.76)	29.95 (26.69, 31.90)	39.27 (35.52, 41.46)	46.90 (42.85, 49.53)	58.37 (53.01, 61.43)	66.90 (61.15, 70.76)	78.70 (70.77, 82.85)	75.59 (65.03, 80.45)	73.39 (60.78, 79.92)	64.24 (50.20, 71.87)	60.11 (43.83, 69.04)
2011	1.57 (1.38, 1.77)	2.64 (2.33, 2.96)	5.11 (4.36, 5.84)	7.26 (6.13, 8.13)	7.93 (6.72, 8.83)	10.33 (8.98, 11.30)	13.85 (12.02, 15.08)	19.88 (17.65, 21.47)	29.78 (26.51, 31.81)	39.19 (35.45, 41.68)	46.73 (42.51, 49.49)	57.73 (52.32, 60.99)	66.33 (60.65, 70.22)	78.59 (70.19, 83.13)	75.60 (64.53, 80.80)	73.63 (60.52, 80.38)	64.99 (50.85, 72.94)	61.47 (44.76, 70.50)
2012	1.53 (1.34, 1.71)	2.59 (2.28, 2.90)	4.97 (4.20, 5.71)	6.98 (5.92, 7.81)	7.73 (6.65, 8.61)	10.18 (8.94, 11.09)	13.74 (11.90, 14.97)	19.63 (17.49, 21.19)	29.53 (26.35, 31.56)	39.28 (35.50, 41.72)	46.52 (42.41, 49.38)	56.83 (51.51, 59.85)	65.97 (60.56, 69.51)	78.20 (69.87, 82.63)	75.36 (64.02, 80.52)	73.72 (60.49, 80.59)	65.63 (51.51, 73.77)	62.06 (45.16, 71.40)
2013	1.50 (1.32, 1.68)	2.57 (2.27, 2.89)	4.89 (4.16, 5.57)	6.88 (5.78, 7.70)	7.68 (6.56, 8.55)	10.21 (8.96, 11.15)	13.77 (11.83, 14.98)	19.44 (17.20, 20.92)	29.46 (26.14, 31.46)	39.07 (35.01, 41.54)	45.88 (41.74, 48.52)	55.63 (50.85, 58.61)	65.22 (59.81, 68.98)	77.74 (69.25, 82.08)	75.63 (64.12, 80.81)	74.01 (60.58, 80.61)	66.17 (51.80, 74.29)	62.87 (45.82, 72.21)
2014	1.50 (1.31, 1.68)	2.54 (2.23, 2.87)	4.75 (4.05, 5.48)	6.60 (5.63, 7.49)	7.54 (6.50, 8.42)	10.11 (8.85, 11.06)	13.70 (11.84, 14.92)	18.98 (16.88, 20.48)	29.34 (26.07, 31.52)	39.00 (35.30, 41.59)	45.40 (41.33, 48.15)	54.30 (50.01, 57.18)	63.77 (58.66, 67.54)	76.95 (68.62, 81.31)	75.66 (64.20, 81.02)	73.45 (60.24, 80.24)	66.02 (51.66, 74.13)	63.40 (45.79, 72.74)
2015	1.48 (1.29, 1.67)	2.49 (2.19, 2.88)	4.73 (4.04, 5.48)	6.47 (5.51, 7.48)	7.46 (6.52, 8.36)	10.13 (8.93, 11.07)	13.63 (11.85, 14.85)	18.85 (16.78, 20.41)	29.34 (26.13, 31.76)	39.45 (35.53, 42.03)	45.59 (41.50, 48.35)	54.29 (50.10, 57.31)	63.48 (58.71, 67.39)	76.31 (69.12, 80.81)	74.79 (63.44, 80.10)	72.91 (59.83, 79.76)	65.82 (51.36, 74.00)	64.00 (45.79, 73.65)
2016	1.45 (1.27, 1.64)	2.46 (2.13, 2.83)	4.80 (4.09, 5.58)	6.49 (5.51, 7.45)	7.47 (6.51, 8.44)	10.18 (8.97, 11.23)	13.71 (12.00, 15.04)	18.82 (16.79, 20.38)	29.01 (25.75, 31.37)	39.45 (35.32, 42.24)	45.48 (41.64, 48.49)	54.26 (50.11, 57.60)	63.42 (58.43, 67.17)	75.47 (68.26, 79.99)	73.95 (62.77, 79.48)	72.26 (59.25, 79.05)	65.48 (50.78, 73.89)	64.21 (45.75, 74.06)
2017	1.42 (1.24, 1.61)	2.46 (2.14, 2.86)	4.90 (4.18, 5.73)	6.57 (5.55, 7.61)	7.49 (6.52, 8.50)	10.23 (8.97, 11.30)	13.80 (11.97, 15.25)	18.68 (16.41, 20.38)	28.67 (25.38, 31.28)	39.04 (34.77, 41.94)	45.24 (41.12, 48.54)	53.92 (49.46, 57.48)	62.99 (57.67, 67.34)	74.70 (67.18, 79.25)	73.31 (62.50, 78.83)	71.41 (58.51, 78.11)	64.32 (49.62, 72.71)	63.00 (44.63, 73.02)
2018	1.43 (1.26, 1.64)	2.53 (2.20, 2.90)	5.10 (4.30, 5.95)	6.79 (5.72, 7.88)	7.58 (6.56, 8.57)	10.37 (9.07, 11.58)	13.88 (12.13, 15.37)	18.63 (16.50, 20.45)	28.55 (25.21, 31.42)	38.57 (34.75, 41.89)	45.41 (41.51, 48.87)	53.73 (49.31, 57.62)	62.80 (57.58, 67.52)	74.73 (66.72, 80.05)	73.51 (62.84, 79.19)	71.43 (58.50, 78.82)	64.07 (49.36, 72.53)	62.68 (44.15, 72.81)
2019	1.42 (1.25, 1.63)	2.53 (2.20, 2.90)	5.20 (4.34, 6.17)	6.93 (5.83, 8.08)	7.61 (6.56, 8.62)	10.42 (9.14, 11.65)	13.90 (12.15, 15.50)	18.67 (16.67, 20.57)	28.47 (25.18, 31.26)	38.19 (34.26, 41.64)	45.74 (41.56, 49.25)	53.83 (49.29, 57.84)	62.57 (57.52, 67.30)	74.69 (66.98, 80.27)	73.33 (62.69, 79.71)	71.67 (58.52, 79.08)	64.00 (48.85, 72.59)	62.40 (44.06, 72.38)
2020	1.41 (1.24, 1.63)	2.54 (2.21, 2.92)	5.32 (4.43, 6.32)	7.02 (5.89, 8.22)	7.64 (6.58, 8.68)	10.50 (9.19, 11.81)	13.98 (12.24, 15.66)	18.57 (16.55, 20.57)	28.23 (24.90, 31.22)	37.94 (34.06, 41.63)	45.70 (41.57, 49.43)	53.63 (49.03, 57.93)	62.29 (57.14, 67.35)	74.13 (66.28, 79.99)	72.86 (62.48, 79.30)	71.10 (58.04, 78.71)	63.33 (48.13, 71.98)	61.94 (43.44, 72.10)
2021	1.40 (1.23, 1.63)	2.56 (2.22, 2.94)	5.46 (4.52, 6.53)	7.17 (6.00, 8.42)	7.69 (6.60, 8.75)	10.58 (9.26, 11.98)	14.05 (12.32, 15.81)	18.54 (16.55, 20.65)	28.05 (24.71, 31.17)	37.57 (33.76, 41.50)	45.83 (41.64, 49.72)	53.58 (48.87, 58.13)	62.08 (56.90, 67.40)	73.85 (65.88, 80.07)	72.58 (62.40, 79.32)	70.88 (57.79, 78.70)	62.85 (47.48, 71.59)	61.43 (42.94, 71.65)
2022	1.39 (1.22, 1.63)	2.58 (2.24, 2.96)	5.60 (4.61, 6.73)	7.32 (6.11, 8.62)	7.74 (6.63, 8.83)	10.67 (9.33, 12.14)	14.12 (12.40, 15.97)	18.51 (16.56, 20.73)	27.87 (24.51, 31.11)	37.20 (33.45, 41.37)	45.96 (41.71, 50.02)	53.52 (48.70, 58.33)	61.86 (56.67, 67.46)	73.57 (65.49, 80.15)	72.31 (62.31, 79.34)	70.65 (57.53, 78.70)	62.38 (46.84, 71.19)	60.93 (42.43, 71.20)
2023	1.38 (1.22, 1.63)	2.60 (2.26, 2.97)	5.74 (4.70, 6.93)	7.47 (6.22, 8.83)	7.80 (6.65, 8.90)	10.76 (9.40, 12.30)	14.19 (12.48, 16.13)	18.48 (16.56, 20.80)	27.69 (24.32, 31.05)	36.84 (33.14, 41.24)	46.09 (41.79, 50.32)	53.47 (48.53, 58.53)	61.65 (56.44, 67.51)	73.28 (65.10, 80.24)	72.03 (62.23, 79.36)	70.43 (57.27, 78.70)	61.90 (46.19, 70.80)	60.42 (41.92, 70.76)
20	1.37 (1.21,	2.62 (2.27,	5.88 (4.79,	7.62 (6.33,	7.85 (6.67,	10.85 (9.46,	14.26 (12.56,	18.44 (16.57,	27.51 (24.13,	36.47 (32.83,	46.21 (41.86,	53.41 (48.37,	61.43 (56.21,	73.00 (64.70,	71.76 (62.14,	70.21 (57.02,	61.42 (45.54,	59.92 (41.41,

24	1.63)	2.99)	7.14)	9.03)	8.97)	12.46)	16.29)	20.88)	31.00)	41.11)	50.62)	58.73)	67.56)	80.32)	79.38)	78.70)	70.40)	70.31)
2025	1.37 (1.20, 1.63)	2.64 (2.29, 3.01)	6.02 (4.88, 7.34)	7.77 (6.44, 9.24)	7.90 (6.69, 9.04)	10.94 (9.53, 12.62)	14.33 (12.64, 16.45)	18.41 (16.57, 20.95)	27.33 (23.93, 30.94)	36.10 (32.52, 40.98)	46.34 (41.94, 50.91)	53.35 (48.20, 58.93)	61.22 (55.98, 67.61)	72.72 (64.31, 80.40)	71.48 (62.06, 79.39)	69.98 (56.76, 78.70)	60.95 (44.90, 70.01)	59.41 (40.90, 69.86)
2026	1.36 (1.20, 1.63)	2.66 (2.31, 3.03)	6.16 (4.97, 7.54)	7.92 (6.55, 9.44)	7.95 (6.71, 9.11)	11.03 (9.60, 12.79)	14.40 (12.72, 16.61)	18.38 (16.58, 21.03)	27.15 (23.74, 30.89)	35.73 (32.22, 40.85)	46.47 (42.01, 51.21)	53.30 (48.04, 59.13)	61.00 (55.74, 67.66)	72.44 (63.92, 80.49)	71.21 (61.97, 79.41)	69.76 (56.51, 78.70)	60.47 (44.25, 69.61)	58.91 (40.39, 69.42)
2027	1.35 (1.19, 1.63)	2.68 (2.32, 3.05)	6.30 (5.06, 7.75)	8.08 (6.66, 9.65)	8.01 (6.73, 9.18)	11.11 (9.67, 12.95)	14.46 (12.80, 16.77)	18.35 (16.58, 21.10)	26.97 (23.55, 30.83)	35.36 (31.91, 40.72)	46.60 (42.09, 51.51)	53.24 (47.87, 59.33)	60.79 (55.51, 67.71)	72.16 (63.53, 80.57)	70.93 (61.88, 79.43)	69.54 (56.25, 78.69)	60.00 (43.61, 69.22)	58.40 (39.89, 68.97)
2028	1.34 (1.18, 1.63)	2.70 (2.34, 3.07)	6.44 (5.15, 7.95)	8.23 (6.77, 9.85)	8.06 (6.76, 9.26)	11.20 (9.74, 13.11)	14.53 (12.88, 16.93)	18.32 (16.59, 21.18)	26.79 (23.35, 30.78)	34.99 (31.60, 40.59)	46.72 (42.16, 51.80)	53.19 (47.71, 59.53)	60.57 (55.28, 67.76)	71.88 (63.13, 80.65)	70.66 (61.80, 79.45)	69.31 (55.99, 78.69)	59.52 (42.96, 68.82)	57.90 (39.38, 68.52)
2029	1.33 (1.18, 1.63)	2.72 (2.36, 3.09)	6.58 (5.23, 8.16)	8.38 (6.88, 10.06)	8.11 (6.78, 9.33)	11.29 (9.80, 13.27)	14.60 (12.96, 17.09)	18.29 (16.59, 21.26)	26.61 (23.16, 30.72)	34.63 (31.29, 40.46)	46.85 (42.23, 52.10)	53.13 (47.54, 59.73)	60.35 (55.05, 67.81)	71.60 (62.74, 80.73)	70.38 (61.71, 79.47)	69.09 (55.74, 78.69)	59.04 (42.32, 68.43)	57.39 (38.87, 68.07)
2030	1.32 (1.17, 1.63)	2.74 (2.37, 3.11)	6.72 (5.32, 8.36)	8.53 (6.99, 10.26)	8.16 (6.80, 9.40)	11.38 (9.87, 13.43)	14.67 (13.04, 17.25)	18.25 (16.60, 21.33)	26.43 (22.96, 30.67)	34.26 (30.99, 40.33)	46.98 (42.31, 52.40)	53.07 (47.37, 59.93)	60.14 (54.81, 67.86)	71.31 (62.35, 80.82)	70.11 (61.63, 79.49)	68.87 (55.48, 78.69)	58.57 (41.67, 68.03)	56.89 (38.36, 67.63)

Table S5. The EAPC of Thyroid cancer Incidence, Deaths, and Disability-Adjusted Life Years Between 1990 and 2030, by countries.

	DALYs (Disability-Adjusted Life Years)	Deaths	Incidence
location	EAPC (95% CI)	EAPC (95% CI)	EAPC (95% CI)
Afghanistan	-0.24 (0.57, -0.41)	-0.07 (0.91, -0.25)	1.31 (1.84, 1.35)
Albania	0.12 (0.42, -0.16)	0.02 (0.17, -0.45)	1.34 (1.53, 1.14)
Algeria	0.10 (0.02, 0.94)	-0.20 (-1.17, 0.73)	2.37 (2.28, 2.79)
American Samoa	0.25 (-0.49, 1.27)	0.20 (-0.62, 1.26)	0.28 (-0.55, 1.29)
Andorra	-0.15 (-1.35, 0.14)	-0.20 (-0.78, 0.12)	-0.03 (-1.04, 0.43)
Angola	0.43 (-0.21, 0.19)	0.56 (0.01, 0.24)	1.95 (1.56, 1.75)
Antigua and Barbuda	-1.31 (-2.09, -0.26)	-1.63 (-1.67, -0.95)	0.36 (-0.71, 1.50)
Argentina	0.08 (-0.42, 0.62)	-0.17 (-0.47, 0.15)	1.39 (-4.85, 4.40)
Armenia	-4.03 (-9.76, -0.33)	-4.90 (-9.47, -1.09)	-1.63 (-6.32, 1.26)
Australia	-0.90 (-1.32, 0.13)	-0.78 (-1.04, -0.40)	-1.06 (-9.35, 3.00)
Austria	-0.62 (-1.30, 0.26)	-0.57 (-1.18, 0.07)	-0.34 (-6.54, 3.01)
Azerbaijan	-0.56 (-1.97, 0.77)	-1.33 (-2.21, -0.75)	1.22 (-0.64, 2.11)
Bahamas	-0.27 (-1.95, 1.51)	-0.38 (-1.85, 1.24)	0.41 (-1.33, 2.01)
Bahrain	-0.19 (-0.19, 0.83)	-0.60 (0.46, 0.42)	1.60 (0.63, 2.36)
Bangladesh	0.64 (-0.51, 1.21)	1.04 (0.28, 1.77)	2.99 (1.98, 3.34)
Barbados	0.29 (-1.12, 1.56)	0.47 (-0.75, 1.35)	0.48 (-0.59, 1.86)
Belarus	0.76 (-4.80, 3.57)	-0.03 (-4.33, 3.01)	1.97 (-2.93, 4.55)
Belgium	-0.54 (-1.04, 0.40)	-0.73 (-0.84, -0.19)	0.07 (-5.84, 3.07)
Belize	-0.09 (-2.37, 1.45)	-0.16 (-2.30, 1.29)	0.81 (-1.06, 1.93)
Benin	-0.99 (-0.30, -0.85)	-0.91 (-0.14, -0.94)	0.36 (0.85, 0.64)
Bermuda	-0.95 (-1.90, 0.85)	-1.32 (-1.85, 0.12)	-0.11 (-1.51, 0.96)

Bhutan	0.26 (-0.08, 1.29)	0.37 (0.09, 1.01)	2.22 (1.96, 2.30)
Bolivia (Plurinational State of)	0.21 (-0.08, 0.05)	0.32 (0.04, 0.45)	1.99 (1.56, 1.95)
Bosnia and Herzegovina	-0.36 (-2.10, 3.07)	-0.42 (-1.90, 2.97)	0.63 (-0.42, 3.77)
Botswana	0.10 (-0.11, 0.35)	0.07 (-0.21, 0.20)	1.43 (0.84, 1.35)
Brazil	-1.66 (-2.03, -1.09)	-1.61 (-2.11, -0.90)	-0.60 (-1.11, -0.15)
Brunei Darussalam	-0.78 (-1.68, -0.11)	-1.39 (-1.85, -0.42)	0.77 (-0.54, 1.66)
Bulgaria	0.79 (-5.84, 3.67)	0.79 (-4.59, 3.43)	1.80 (-3.53, 4.28)
Burkina Faso	0.30 (-0.03, 0.64)	0.16 (0.20, 0.37)	1.19 (0.75, 1.43)
Burundi	0.22 (0.26, 0.55)	0.22 (-0.40, 0.40)	0.64 (0.99, 1.29)
Cabo Verde	4.00 (2.99, 4.48)	5.11 (4.50, 5.39)	3.64 (3.35, 4.21)
Cambodia	0.27 (-0.29, 0.61)	0.47 (0.11, 0.61)	2.16 (1.73, 2.54)
Cameroon	-1.00 (-1.73, -1.13)	-0.96 (-1.41, -0.89)	1.28 (1.58, 1.55)
Canada	-0.17 (-0.83, 0.44)	0.07 (-0.18, 0.35)	-0.21 (-8.01, 3.18)
Central African Republic	-0.92 (-0.72, -0.47)	-0.75 (-0.52, 0.08)	-0.68 (-0.17, -0.80)
Chad	-0.61 (-1.24, -0.23)	-0.73 (-0.65, -0.24)	0.25 (0.34, 0.33)
Chile	-0.61 (-1.21, 0.08)	-0.70 (-1.19, 0.01)	0.07 (-7.62, 3.51)
China	-0.63 (-1.68, -0.00)	-1.23 (-2.01, -0.18)	2.06 (0.94, 2.69)
Colombia	-0.94 (-11.11, 2.54)	-0.88 (-8.90, 2.50)	0.46 (-7.76, 3.82)
Comoros	-2.15 (-6.83, -1.07)	-0.97 (-2.86, -0.56)	-0.99 (-5.49, 0.60)
Congo	-0.52 (-0.94, -0.10)	-0.39 (-0.31, 0.04)	0.63 (0.74, 0.96)
Cook Islands	0.16 (-1.31, 0.96)	-0.01 (-0.88, 0.70)	1.70 (-0.12, 2.75)
Costa Rica	0.45 (-6.95, 3.63)	0.25 (-6.05, 3.03)	1.41 (-5.03, 4.25)
Croatia	-0.98 (-9.32, 2.78)	-1.32 (-7.95, 2.12)	-0.37 (-8.40, 3.14)
Cuba	-0.73 (-6.93, 2.96)	-1.02 (-6.32, 2.30)	0.59 (-4.27, 3.68)

Cyprus	-0.41 (-1.02, 0.66)	-1.16 (-1.92, -0.17)	0.11 (-1.04, 1.08)
Czechia	0.09 (-5.58, 2.66)	-0.13 (-5.64, 2.55)	0.71 (-5.72, 3.22)
Côte d'Ivoire	-1.24 (-1.21, -0.63)	-1.06 (-0.52, -0.81)	0.23 (-0.16, 0.63)
Democratic People's Republic of Korea	-0.93 (-0.61, -0.78)	-1.14 (-0.70, -1.04)	0.60 (0.31, 0.71)
Democratic Republic of the Congo	-0.13 (-0.46, 0.04)	-0.06 (-0.47, -0.16)	1.25 (0.98, 1.33)
Denmark	-0.46 (-1.06, 0.23)	-0.97 (-1.93, -0.52)	0.29 (-4.93, 3.62)
Djibouti	1.12 (1.70, 1.14)	0.99 (1.64, 0.10)	2.79 (2.95, 3.09)
Dominica	-0.31 (-1.18, 0.50)	-0.38 (-0.84, 0.46)	-0.22 (-0.99, 0.87)
Dominican Republic	-0.07 (-5.34, 2.61)	-0.06 (-2.72, 2.28)	1.60 (-2.02, 3.63)
Ecuador	-1.00 (-1.55, 2.71)	-1.37 (-0.88, 2.15)	0.58 (-1.54, 3.75)
Egypt	0.05 (-4.06, -0.01)	-0.84 (-5.07, -0.74)	3.44 (0.94, 4.07)
El Salvador	0.25 (-2.87, 1.75)	0.46 (-2.01, 1.72)	1.50 (-1.20, 2.42)
Equatorial Guinea	0.54 (1.01, 0.11)	0.72 (0.86, -0.36)	1.76 (2.23, 1.19)
Eritrea	0.20 (0.33, 0.00)	0.18 (0.53, -0.30)	1.48 (1.50, 1.66)
Estonia	-0.68 (-7.77, 2.53)	-0.90 (-7.43, 2.13)	-0.49 (-6.21, 2.71)
Eswatini	-0.52 (-0.70, -0.90)	-0.39 (-0.05, -0.56)	0.64 (1.14, 0.06)
Ethiopia	0.27 (0.43, -0.05)	0.38 (0.51, 0.00)	2.20 (2.15, 2.56)
Fiji	0.90 (0.25, 1.98)	1.06 (0.10, 1.84)	2.17 (1.62, 2.89)
Finland	-0.70 (-1.23, 0.12)	-0.97 (-1.13, -0.55)	0.08 (-6.39, 3.26)
France	0.95 (0.07, 1.52)	0.90 (0.26, 1.29)	0.98 (-5.63, 3.83)
Gabon	0.20 (0.07, 0.29)	0.24 (0.00, 0.52)	1.82 (1.82, 2.03)
Gambia	1.27 (1.10, 1.78)	1.21 (1.32, 1.28)	2.36 (1.78, 2.80)
Georgia	-1.94 (-5.42, 0.86)	-2.35 (-4.66, 0.59)	0.11 (-5.69, 2.18)
Germany	-0.52 (-0.72, 0.49)	-0.68 (-0.85, -0.02)	0.08 (-9.13, 4.09)

Ghana	-0.42 (-0.89, -0.02)	-0.12 (-0.00, 0.07)	1.51 (1.09, 1.78)
Greece	0.81 (0.29, 1.32)	1.21 (1.03, 1.33)	0.95 (-5.11, 4.26)
Greenland	-1.45 (0.02, -0.43)	-1.79 (0.36, -1.06)	-0.26 (0.98, 0.35)
Grenada	-3.56 (-5.20, -2.07)	-3.02 (-3.82, -1.85)	-1.48 (-3.14, -0.65)
Guam	0.66 (0.11, 1.89)	0.96 (1.01, 1.98)	1.15 (1.45, 1.94)
Guatemala	-0.66 (-4.83, 2.11)	-0.53 (-3.83, 1.73)	0.75 (-2.50, 2.70)
Guinea	-1.23 (-1.83, -0.24)	-1.18 (-2.25, -0.98)	0.25 (-0.40, 1.02)
Guinea-Bissau	-1.56 (-0.90, -1.27)	-1.30 (-1.17, -1.09)	-0.38 (-0.10, 0.28)
Guyana	-0.11 (-1.46, 1.16)	-0.20 (-1.72, 1.02)	1.57 (0.40, 2.59)
Haiti	-0.51 (-0.22, -0.33)	-0.36 (-0.28, 0.05)	0.34 (0.92, 0.14)
Honduras	-0.65 (-1.25, 0.03)	-0.98 (-1.51, -0.33)	0.92 (0.73, 0.99)
Hungary	-0.00 (-5.64, 2.78)	-0.43 (-5.70, 2.67)	1.29 (-3.55, 3.69)
Iceland	-0.71 (-1.43, -0.18)	-0.91 (-1.26, -0.29)	-0.29 (-1.81, 0.75)
India	0.74 (-0.15, 1.79)	0.64 (0.37, 1.74)	2.50 (1.67, 3.77)
Indonesia	-0.38 (-0.07, 0.19)	-0.33 (-0.25, 0.20)	1.24 (1.45, 1.79)
Iran (Islamic Republic of)	-0.30 (0.65, 0.10)	-0.51 (0.35, 0.15)	2.00 (2.73, 2.26)
Iraq	-1.26 (-0.42, -1.93)	-0.60 (-0.11, -1.55)	0.96 (1.32, 1.02)
Ireland	-1.51 (-2.14, -0.34)	-1.65 (-2.47, -1.04)	-1.04 (-9.77, 2.78)
Israel	-0.11 (-0.63, 0.45)	-0.29 (-0.39, -0.04)	0.44 (-6.09, 3.69)
Italy	0.17 (-0.28, 0.40)	0.01 (-0.14, 0.22)	0.40 (-5.13, 3.25)
Jamaica	0.16 (-3.20, 2.08)	0.33 (-2.32, 1.94)	0.42 (-2.72, 2.55)
Japan	-0.88 (-1.05, -0.35)	-0.97 (-0.94, -0.73)	-0.90 (-6.14, 2.12)
Jordan	0.80 (0.64, 1.69)	0.50 (0.24, 1.57)	1.86 (1.33, 2.36)
Kazakhstan	-0.69 (-4.25, 1.15)	-1.27 (-4.80, 0.26)	1.15 (-1.33, 2.60)

Kenya	-0.41 (-1.23, -0.01)	-0.24 (-0.62, 0.02)	1.74 (1.22, 1.77)
Kiribati	-0.06 (-0.43, 0.46)	-0.03 (-1.17, -0.95)	1.10 (0.19, 1.68)
Kuwait	1.69 (0.22, 2.75)	1.64 (0.66, 2.49)	2.76 (1.48, 3.64)
Kyrgyzstan	-1.59 (-3.89, 0.83)	-1.89 (-3.64, 0.26)	0.46 (-1.75, 2.10)
Lao People's Democratic Republic	-0.25 (-0.02, -0.29)	0.04 (0.47, 0.50)	1.45 (1.34, 1.37)
Latvia	-0.56 (-3.86, 1.62)	-1.16 (-4.49, 1.00)	1.01 (-2.48, 3.03)
Lebanon	0.29 (-0.72, 0.76)	-0.34 (-0.58, 0.06)	1.68 (0.28, 2.95)
Lesotho	-1.45 (-1.42, -1.09)	-1.42 (-1.82, -1.19)	-0.19 (0.37, 0.20)
Liberia	-0.77 (-1.09, -0.29)	-0.81 (-0.65, -0.03)	0.28 (-0.06, 0.49)
Libya	0.18 (-0.61, 1.52)	0.22 (-0.39, 0.16)	0.26 (-0.75, 1.43)
Lithuania	-1.31 (-9.42, 2.01)	-1.69 (-9.57, 1.52)	0.06 (-6.38, 3.36)
Luxembourg	-0.30 (-1.04, 0.51)	-0.43 (-1.19, 0.66)	-0.34 (-2.85, 1.17)
Madagascar	0.28 (0.48, -0.30)	0.39 (0.15, 0.42)	1.55 (1.75, 1.19)
Malawi	0.00 (-0.87, 0.85)	-0.01 (-0.24, 0.71)	1.31 (1.00, 2.01)
Malaysia	1.25 (-1.55, 2.24)	0.76 (-2.37, 1.17)	2.86 (0.34, 4.03)
Maldives	1.30 (0.00, 2.30)	1.15 (0.04, 2.27)	2.30 (1.52, 2.95)
Mali	0.11 (-0.19, 1.10)	-0.10 (-0.18, 0.75)	1.32 (0.63, 1.90)
Malta	-0.09 (-1.61, 0.78)	-0.53 (-1.74, 0.03)	1.05 (-1.12, 2.26)
Marshall Islands	-0.24 (-0.71, 0.39)	-0.35 (-1.24, 0.16)	0.87 (1.18, 1.50)
Mauritania	-1.17 (-3.92, -0.57)	-0.84 (-1.79, -0.14)	0.71 (-0.97, 1.46)
Mauritius	-0.18 (-5.41, 3.13)	-0.35 (-4.53, 2.88)	1.39 (-3.83, 4.16)
Mexico	0.41 (-2.80, 3.06)	0.26 (-2.11, 2.97)	1.65 (-2.93, 4.44)
Micronesia (Federated States of)	0.39 (-0.10, 0.77)	0.41 (-0.12, 0.82)	1.52 (0.74, 1.67)
Monaco	-1.24 (-1.97, 0.07)	-1.20 (-2.73, -0.65)	-1.03 (-2.77, 0.28)

Mongolia	0.41 (0.71, 0.83)	0.42 (0.17, 0.70)	1.68 (2.13, 2.26)
Montenegro	-0.52 (-3.37, 1.02)	-1.12 (-3.13, 0.27)	0.52 (-1.52, 1.94)
Morocco	-0.23 (-0.40, 0.22)	-0.21 (-0.40, -0.77)	2.09 (1.81, 2.98)
Mozambique	-0.16 (-1.40, 0.68)	-0.01 (-1.05, 0.92)	1.19 (-0.07, 1.94)
Myanmar	-0.48 (-0.22, 0.00)	-0.34 (-0.05, 0.22)	1.73 (1.81, 2.49)
Namibia	0.04 (-0.35, 0.26)	0.22 (-0.49, 0.89)	1.54 (1.48, 1.60)
Nauru	-0.51 (-1.11, -0.34)	-0.98 (-1.84, -0.99)	1.87 (1.53, 1.85)
Nepal	0.64 (0.13, 1.05)	0.93 (0.09, 1.20)	2.34 (1.78, 2.46)
Netherlands	-0.75 (-0.71, 0.14)	-1.01 (-1.29, -0.37)	-0.24 (-6.80, 3.47)
New Zealand	0.04 (0.13, 0.31)	-0.02 (0.53, 0.08)	0.30 (-5.41, 2.93)
Nicaragua	-0.39 (-2.67, 2.05)	-0.31 (-1.39, 1.24)	0.84 (-1.39, 3.15)
Niger	-0.13 (-0.44, 0.18)	-0.25 (-0.65, 0.27)	0.70 (0.28, 0.87)
Nigeria	-0.90 (-1.67, -0.69)	-0.69 (-1.61, -0.47)	0.34 (-0.22, 0.76)
Niue	-0.28 (-0.44, -0.07)	-0.35 (-0.55, -0.12)	0.51 (0.20, 0.93)
North Macedonia	-0.02 (-4.60, 3.08)	-0.11 (-4.38, 2.16)	0.76 (-1.42, 3.79)
Northern Mariana Islands	-0.39 (-0.60, 0.40)	-0.53 (-1.01, 0.12)	1.13 (0.69, 1.77)
Norway	-1.48 (-1.83, -0.98)	-1.51 (-1.29, -1.02)	-1.48 (-6.48, 1.30)
Oman	-2.94 (-3.92, -1.43)	-4.93 (-5.12, -3.81)	0.45 (0.17, 1.10)
Pakistan	-0.53 (-0.22, -0.50)	-0.47 (-0.41, -0.29)	1.36 (1.35, 2.15)
Palau	0.24 (0.15, 0.56)	0.04 (-0.25, 0.22)	1.31 (0.74, 1.41)
Palestine	0.76 (-0.37, 2.08)	0.65 (-0.70, 1.90)	2.87 (2.18, 3.80)
Panama	0.02 (-8.46, 3.69)	-0.46 (-8.57, 3.06)	2.22 (-3.30, 4.95)
Papua New Guinea	0.13 (0.02, 0.13)	0.05 (-0.11, -0.50)	1.11 (1.04, 1.39)
Paraguay	0.17 (-0.30, 3.04)	-0.35 (0.09, 2.37)	2.12 (1.35, 4.45)

Peru	-0.69 (-2.84, 1.19)	-0.98 (-2.35, 1.01)	1.10 (-0.29, 2.77)
Philippines	-0.74 (-3.64, 1.06)	-0.78 (-3.46, 0.77)	0.83 (-1.72, 2.56)
Poland	-0.08 (-3.92, 0.52)	-0.25 (-4.02, 0.32)	0.68 (-4.38, 2.27)
Portugal	-0.32 (-0.99, 0.39)	-0.54 (-0.82, -0.10)	0.38 (-7.59, 3.47)
Puerto Rico	-0.24 (-7.35, 3.32)	-0.33 (-5.93, 3.15)	0.16 (-8.09, 3.47)
Qatar	-2.59 (-3.75, -1.06)	-3.90 (-5.33, -2.42)	-0.41 (-1.52, 0.83)
Republic of Korea	-2.58 (1.00, -1.66)	-1.73 (0.60, -0.81)	-3.31 (1.87, -0.08)
Republic of Moldova	-4.39 (-8.49, -0.55)	-4.20 (-9.92, -0.66)	-2.27 (-6.37, 0.64)
Romania	-0.15 (-7.00, 3.23)	-0.46 (-6.42, 2.88)	1.15 (-3.74, 3.85)
Russian Federation	-1.53 (-6.43, 0.83)	-1.67 (-6.60, 1.38)	-0.75 (-9.86, 2.94)
Rwanda	0.63 (0.19, 0.72)	0.77 (0.55, 1.14)	2.18 (1.85, 2.22)
Saint Kitts and Nevis	1.97 (1.44, 2.53)	1.90 (1.64, 2.13)	2.16 (1.36, 3.18)
Saint Lucia	0.39 (-1.34, 1.86)	0.74 (-0.23, 1.87)	0.95 (-0.52, 1.98)
Saint Vincent and the Grenadines	-1.00 (-2.87, 0.57)	-1.40 (-2.84, 0.09)	0.39 (-1.20, 1.46)
Samoa	0.04 (-0.69, 0.41)	-0.06 (0.02, 0.50)	2.26 (1.34, 2.70)
San Marino	-0.35 (-1.81, 1.11)	-0.62 (-1.11, 0.98)	0.01 (-0.67, 0.73)
Sao Tome and Principe	0.25 (-0.68, 0.78)	0.44 (-0.37, 1.25)	1.94 (1.31, 2.25)
Saudi Arabia	-0.22 (-1.54, 0.79)	-1.37 (-2.44, -0.35)	2.10 (1.34, 2.63)
Senegal	-0.66 (-1.63, 0.01)	-0.45 (-0.93, 0.12)	0.78 (-0.56, 1.40)
Serbia	-0.94 (-3.49, 2.96)	-0.98 (-3.50, 2.45)	-0.22 (-2.98, 3.28)
Seychelles	0.93 (0.05, 1.41)	0.77 (0.08, 1.47)	2.43 (1.69, 2.80)
Sierra Leone	-0.20 (-1.29, 0.08)	-0.39 (-1.29, -0.08)	1.07 (0.56, 1.34)
Singapore	-0.73 (-1.62, 0.43)	-1.19 (-1.82, -0.46)	0.18 (-6.54, 3.08)
Slovakia	0.14 (-4.30, 2.45)	-0.14 (-3.72, 2.49)	0.96 (-2.66, 2.85)

Slovenia	0.28 (-6.24, 3.57)	-0.04 (-4.50, 3.54)	0.94 (-4.66, 3.97)
Solomon Islands	-0.38 (-0.76, -0.46)	-0.21 (-0.32, -0.10)	0.93 (0.70, 1.17)
Somalia	-0.03 (0.18, 0.02)	0.11 (-0.13, 0.22)	0.23 (0.39, -0.21)
South Africa	-5.13 (-7.38, -2.51)	-5.46 (-5.68, -2.98)	-2.32 (-4.14, -1.16)
South Sudan	0.63 (0.45, 0.90)	0.55 (0.68, 0.98)	1.46 (1.34, 1.58)
Spain	-0.18 (-0.39, 0.89)	-0.35 (-0.50, 0.23)	0.38 (-4.80, 3.82)
Sri Lanka	-0.30 (-3.13, 1.18)	-0.96 (-3.33, 0.12)	1.98 (0.06, 3.45)
Sudan	0.65 (0.78, 0.50)	0.72 (0.76, 0.82)	2.92 (3.47, 3.13)
Suriname	0.32 (-1.41, 1.88)	0.10 (-1.44, 1.39)	1.29 (-0.13, 2.60)
Sweden	-1.21 (-1.22, -0.81)	-1.05 (-1.31, -0.46)	-1.07 (-6.56, 1.66)
Switzerland	-0.70 (-0.37, 0.46)	-1.19 (0.08, -0.54)	0.07 (-3.82, 3.72)
Syrian Arab Republic	-0.05 (-0.81, 1.26)	-0.07 (-0.42, 0.99)	1.16 (0.60, 2.07)
Taiwan (Province of China)	0.48 (-5.81, 4.30)	-0.15 (-6.00, 3.67)	1.50 (-4.89, 5.16)
Tajikistan	-1.28 (-1.64, 1.14)	-1.94 (-2.56, 0.01)	0.09 (-0.32, 2.06)
Thailand	0.58 (-4.24, -0.03)	0.40 (-2.95, 0.29)	2.09 (-1.89, 1.42)
Timor-Leste	0.45 (-0.03, 0.57)	0.53 (-0.17, 0.42)	1.02 (1.24, 0.97)
Togo	-0.18 (0.03, 0.21)	-0.18 (-0.04, 0.23)	1.43 (1.74, 1.75)
Tokelau	-0.23 (-0.03, 0.15)	-0.71 (-0.64, -0.65)	1.81 (1.02, 2.25)
Tonga	-0.01 (-0.01, 1.04)	-0.00 (0.07, 0.62)	1.35 (1.24, 1.89)
Trinidad and Tobago	0.29 (-0.29, 0.73)	0.24 (-0.30, 0.36)	1.00 (0.38, 1.39)
Tunisia	-0.14 (-0.31, 0.15)	-0.64 (-0.93, -0.01)	1.59 (1.48, 1.90)
Turkey	-0.50 (-4.92, 0.44)	-0.99 (-5.26, 0.09)	1.65 (-0.82, 2.82)
Turkmenistan	-0.87 (-5.33, 2.14)	-0.61 (-4.42, 2.16)	0.35 (-3.33, 2.88)
Tuvalu	-0.20 (-0.69, 0.05)	-0.03 (0.28, -0.04)	1.17 (1.34, 1.20)

Uganda	-0.63 (-0.55, 0.08)	-0.58 (-0.70, -0.00)	1.32 (1.02, 2.20)
Ukraine	-0.04 (-3.17, 2.13)	0.35 (-3.54, 2.82)	-0.14 (-5.05, 2.41)
United Arab Emirates	0.57 (-0.17, 0.60)	0.26 (0.32, 0.57)	2.34 (1.98, 2.79)
United Kingdom	0.15 (0.02, 0.48)	0.08 (0.09, 0.19)	0.23 (-6.20, 3.01)
United Republic of Tanzania	0.73 (0.22, 0.99)	0.68 (0.40, 0.96)	2.50 (1.97, 2.80)
United States of America	-0.48 (-0.89, -0.36)	-0.16 (-0.04, -0.13)	-0.76 (-6.81, 2.79)
United States Virgin Islands	-0.90 (-2.25, -0.11)	-1.05 (-1.67, -0.75)	-0.89 (-2.19, -0.33)
Uruguay	0.25 (-0.19, 0.53)	0.12 (-0.10, 0.45)	1.36 (-4.79, 4.23)
Uzbekistan	-2.63 (-6.11, 0.18)	-3.33 (-5.72, -0.97)	-0.40 (-3.98, 1.99)
Vanuatu	-0.06 (0.01, -0.28)	-0.01 (-0.39, -0.52)	0.77 (0.89, 0.77)
Venezuela (Bolivarian Republic of)	1.38 (-1.35, 3.34)	1.64 (-0.76, 3.27)	0.93 (-2.23, 2.86)
Viet Nam	-0.49 (-1.26, 0.58)	-0.60 (-2.15, 0.34)	1.79 (0.71, 1.96)
Yemen	1.06 (0.79, 0.88)	0.89 (0.10, 0.75)	1.04 (0.65, 1.47)
Zambia	0.19 (-0.42, 0.97)	0.39 (0.08, 0.61)	2.18 (1.92, 3.01)
Zimbabwe	-0.53 (-0.45, 0.03)	-0.55 (-0.93, -0.03)	0.68 (0.61, 1.33)

Table S6. The Top 10 Countries With the Largest EAPC of Age-Standardized Incidence Rate, Death Rate, and Disability Adjusted Life Years Rate of Thyroid cancer Between 2020 and 2030.

order	Countries	EAPC of ASIR	Countries	EAPC of ASDR	Countries	EAPC of Age-standardized DALY rate
		No.(95%CI)		No.(95%CI)		No.(95%CI)
1	Cabo Verde	3.64 (3.35, 4.21)	Cabo Verde	5.11 (4.50, 5.39)	Cabo Verde	4.00 (2.99, 4.48)
2	Egypt	3.44 (0.94, 4.07)	Saint Kitts and Nevis	1.90 (1.64, 2.13)	Saint Kitts and Nevis	1.97 (1.44, 2.53)
3	Bangladesh	2.99 (1.98, 3.34)	Venezuela (Bolivarian Republic of)	1.64 (-0.76, 3.27)	Kuwait	1.69 (0.22, 2.75)
4	Sudan	2.92 (3.47, 3.13)	Kuwait	1.64 (0.66, 2.49)	Venezuela (Bolivarian Republic of)	1.38 (-1.35, 3.34)
5	Palestine	2.87 (2.18, 3.80)	Greece	1.21 (1.03, 1.33)	Maldives	1.30 (0.00, 2.30)
6	Malaysia	2.86 (0.34, 4.03)	Gambia	1.21 (1.32, 1.28)	Gambia	1.27 (1.10, 1.78)
7	Djibouti	2.79 (2.95, 3.09)	Maldives	1.15 (0.04, 2.27)	Malaysia	1.25 (-1.55, 2.24)
8	Kuwait	2.76 (1.48, 3.64)	Fiji	1.06 (0.10, 1.84)	Djibouti	1.12 (1.70, 1.14)
9	India	2.50 (1.67, 3.77)	Bangladesh	1.04 (0.28, 1.77)	Yemen	1.06 (0.79, 0.88)
10	United Republic of Tanzania	2.50 (1.97, 2.80)	Djibouti	0.99 (1.64, 0.10)	France	0.95 (0.07, 1.52)

Table S7. The Bottom 10 Countries With the Largest EAPC of Age-Standardized Incidence Rate, Death Rate, and Disability Adjusted Life Years Rate of Thyroid cancer Between 2020 and 2030.

order	Countries	EAPC of ASIR	Countries	EAPC of ASDR	Countries	EAPC of Age-standardized DALY rate
		No.(95%CI)		No.(95%CI)		No.(95%CI)
1	Republic of Korea	-3.31 (1.87, -0.08)	South Africa	-5.46 (-5.68, -2.98)	South Africa	-5.13 (-7.38, -2.51)
2	South Africa	-2.32 (-4.14, -1.16)	Oman	-4.93 (-5.12, -3.81)	Republic of Moldova	-4.39 (-8.49, -0.55)
3	Republic of Moldova	-2.27 (-6.37, 0.64)	Armenia	-4.90 (-9.47, -1.09)	Armenia	-4.03 (-9.76, -0.33)
4	Armenia	-1.63 (-6.32, 1.26)	Republic of Moldova	-4.20 (-9.92, -0.66)	Grenada	-3.56 (-5.20, -2.07)
5	Norway	-1.48 (-6.48, 1.30)	Qatar	-3.90 (-5.33, -2.42)	Oman	-2.94 (-3.92, -1.43)
6	Grenada	-1.48 (-3.14, -0.65)	Uzbekistan	-3.33 (-5.72, -0.97)	Uzbekistan	-2.63 (-6.11, 0.18)
7	Sweden	-1.07 (-6.56, 1.66)	Grenada	-3.02 (-3.82, -1.85)	Qatar	-2.59 (-3.75, -1.06)
8	Australia	-1.06 (-9.35, 3.00)	Georgia	-2.35 (-4.66, 0.59)	Republic of Korea	-2.58 (1.00, -1.66)
9	Ireland	-1.04 (-9.77, 2.78)	Tajikistan	-1.94 (-2.56, 0.01)	Comoros	-2.15 (-6.83, -1.07)
10	Monaco	-1.03 (-2.77, 0.28)	Kyrgyzstan	-1.89 (-3.64, 0.26)	Georgia	-1.94 (-5.42, 0.86)

Figure S1. The EAPC in global thyroid cancer burden from 2020 to 2030, by regions. Panel A: ASIR (age-standardized incidence rate); Panel B : ASDR (age-standardized death rate); Panel C: DALY (disability-adjusted life-year).

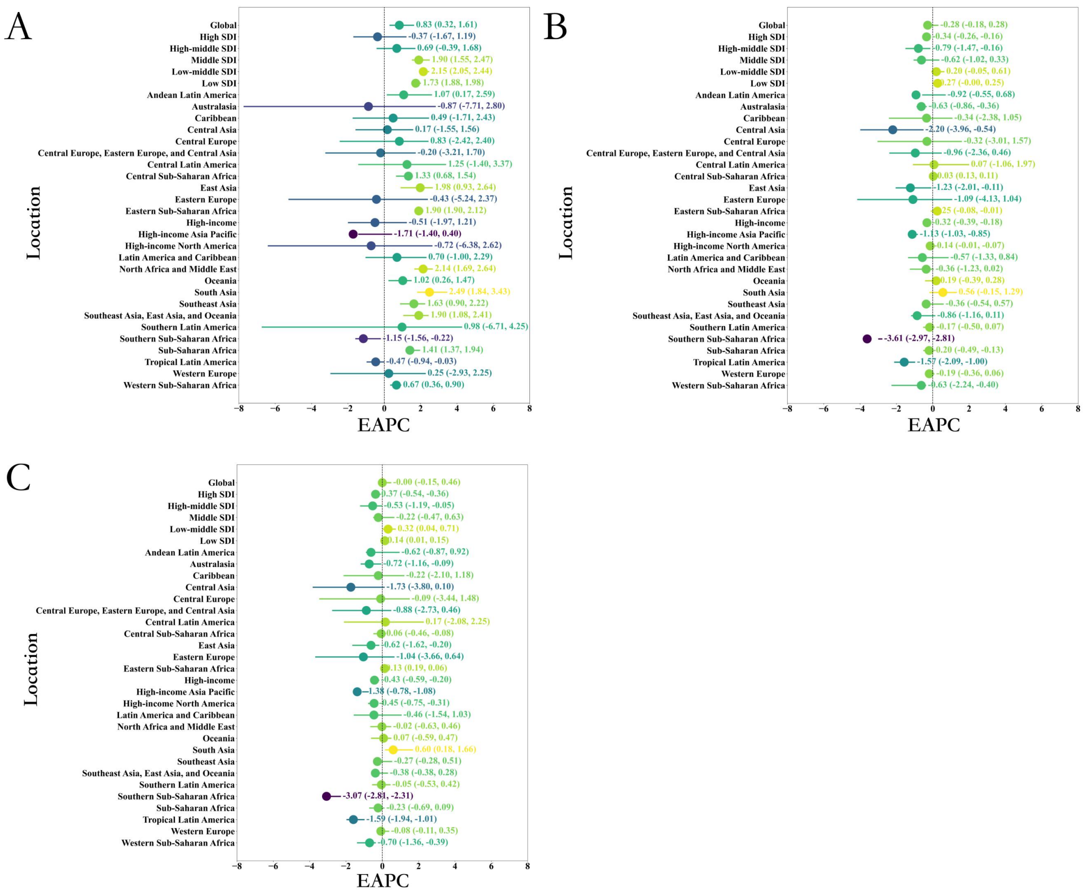

Figure S2. The EAPC in global thyroid cancer burden from 1990 to 2019, by regions. Panel A: ASIR (age-standardized incidence rate); Panel B : ASDR (age-standardized death rate); Panel C: DALY (disability-adjusted life-year).

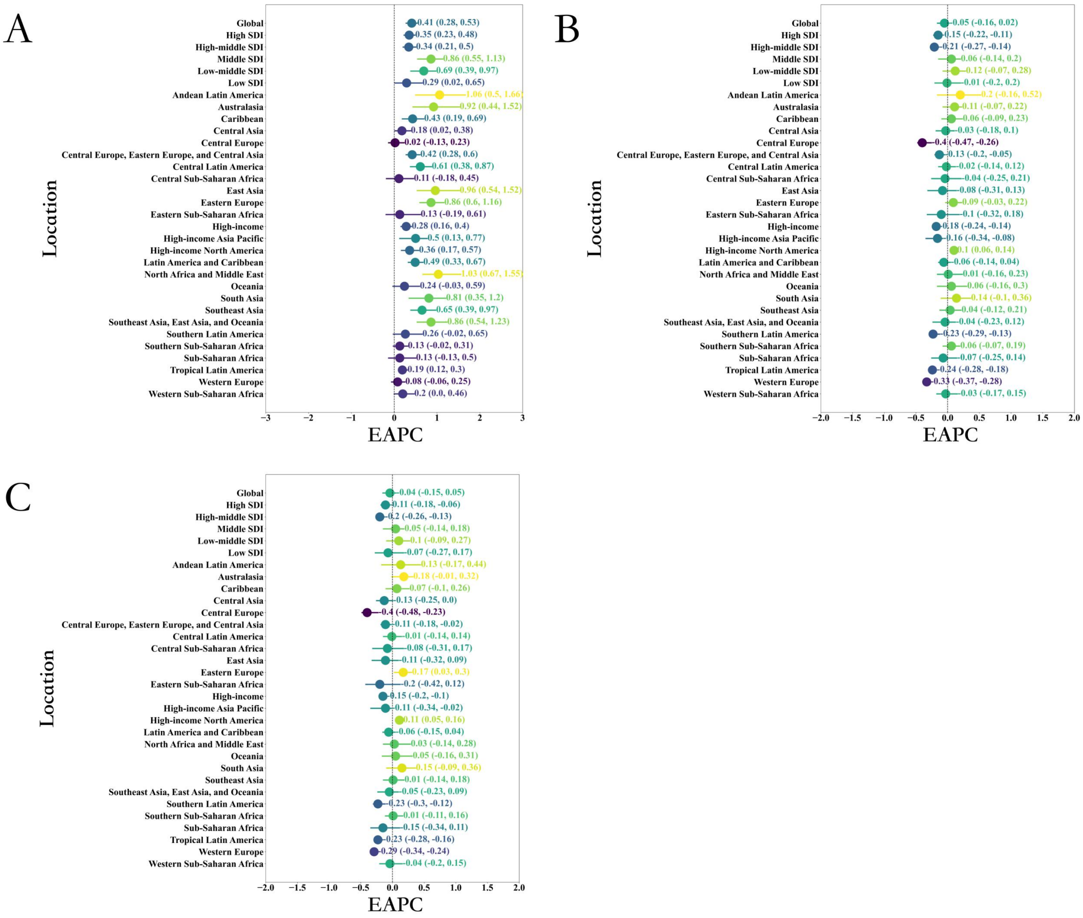

Figure S3. Trends in incidence rate of global thyroid cancer burden from 1990 to 2030, by ages and genders. Panel A: Both; Panel B: Male; Panel C: Female.

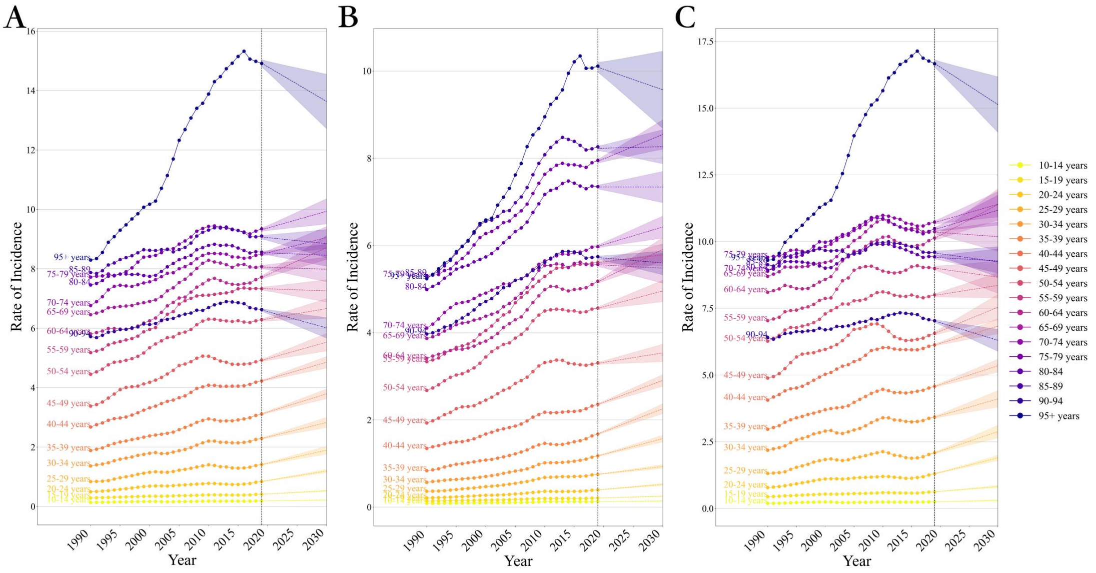

Figure S4. Trends in death rate of global thyroid cancer burden from 1990 to 2030, by ages and genders. Panel A: Both; Panel B: Male; Panel C: Female.

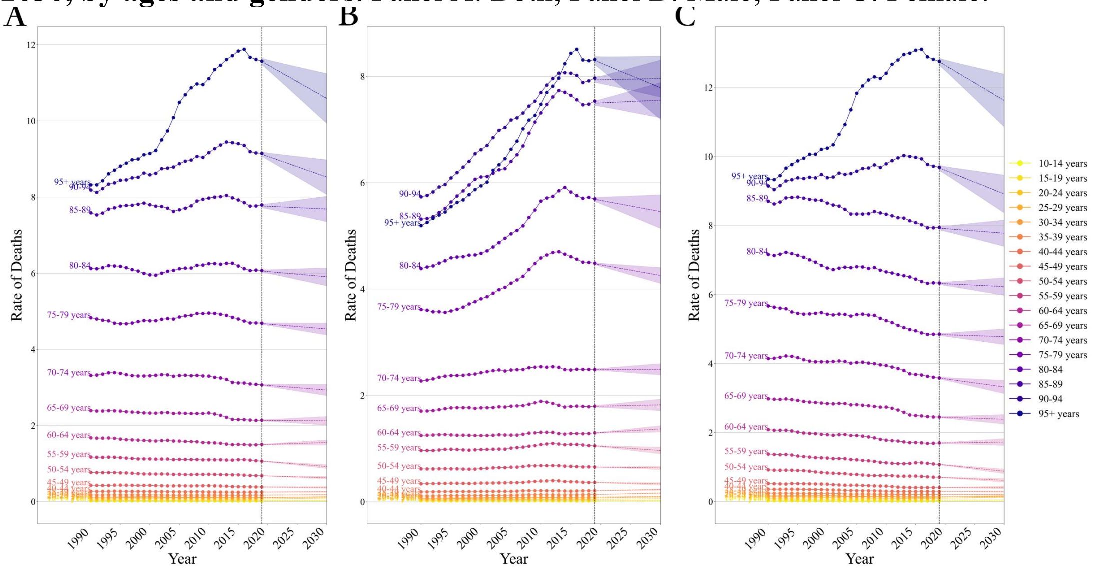

Figure S5. Trends in DALY(disability adjusted life-year rate) of global thyroid cancer burden from 1990 to 2030, by ages and genders. Panel A: Both; Panel B: Male; Panel C: Female.

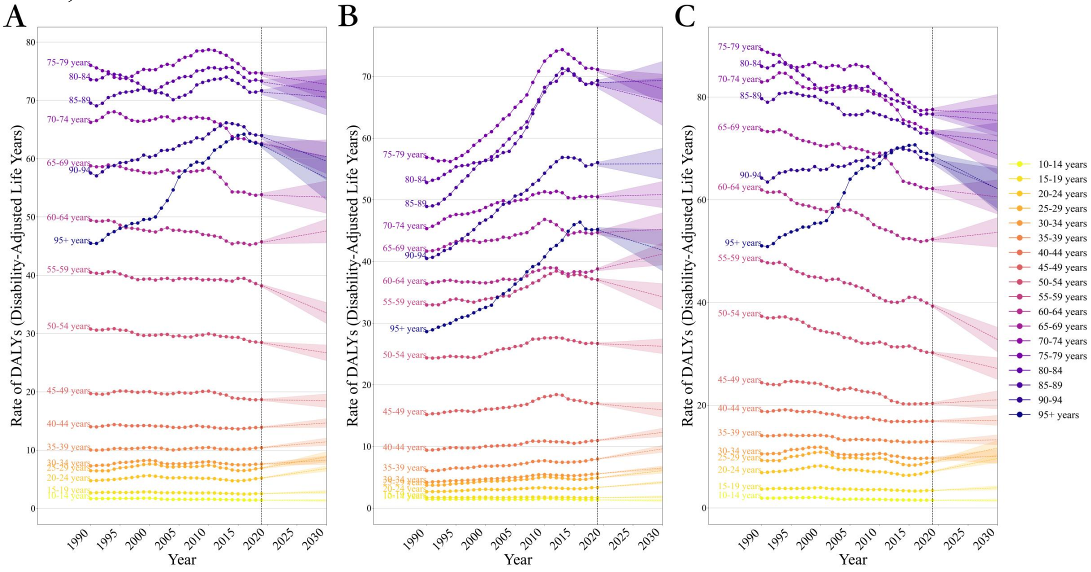

Figure S6. The EAPC of ASIR (age standardized incidence rate) in global thyroid cancer burden from 1990 to 2019, by countries.

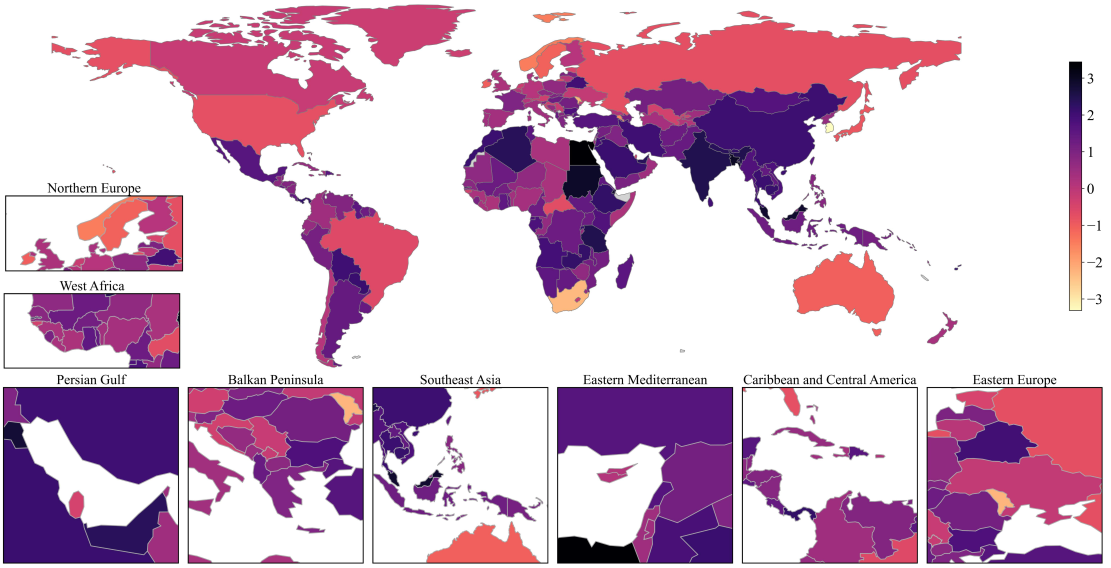

Figure S7. The EAPC of ASDR (age standardized death rate) in global thyroid cancer burden from 1990 to 2019, by countries.

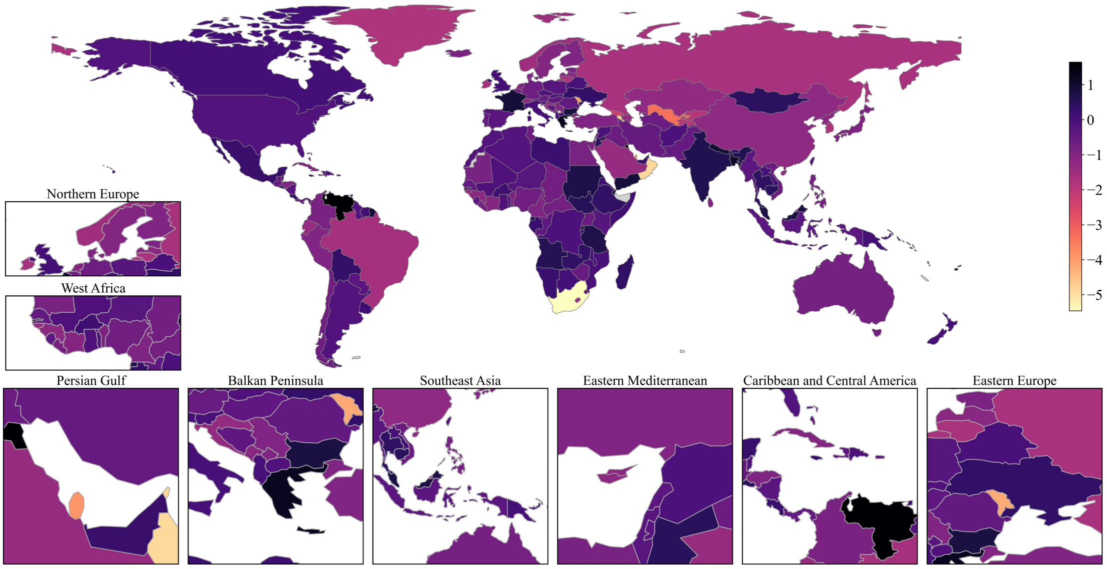

Figure S8. The EAPC of age-standeried DALY (disability adjusted life-year) rate in global thyroid cancer burden from 1990 to 2019, by countries.

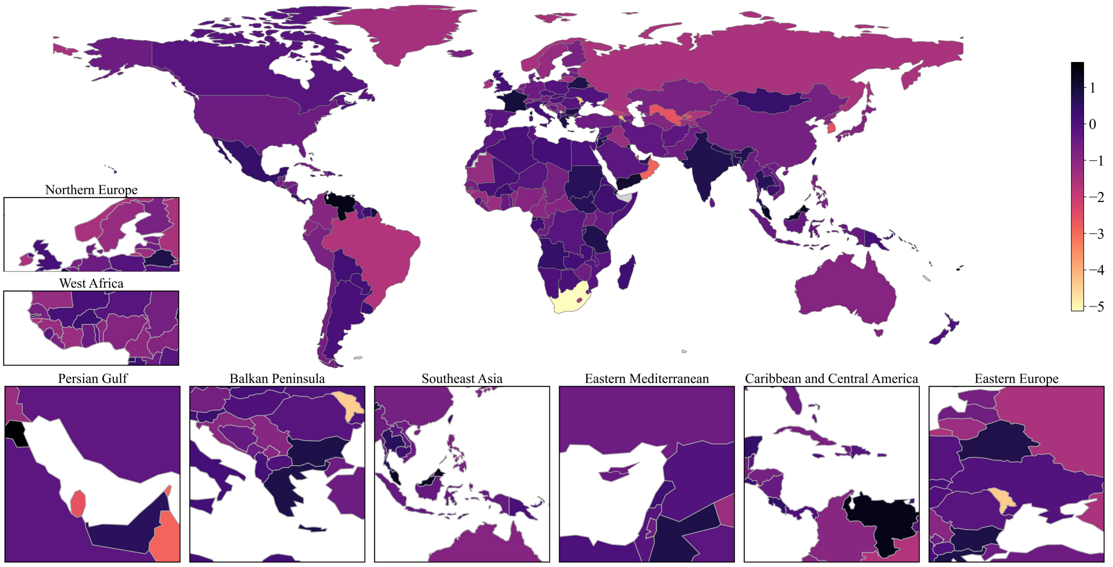

Figure S9. Trends in global thyroid cancer burden from 1990 to 2019 and Projected Rates from 2020 to 2030. Panel A: ASIR (age-standardized incidence rate); Panel B : ASDR (age-standardized death rate); Panel C: DALY (disability-adjusted life-year).

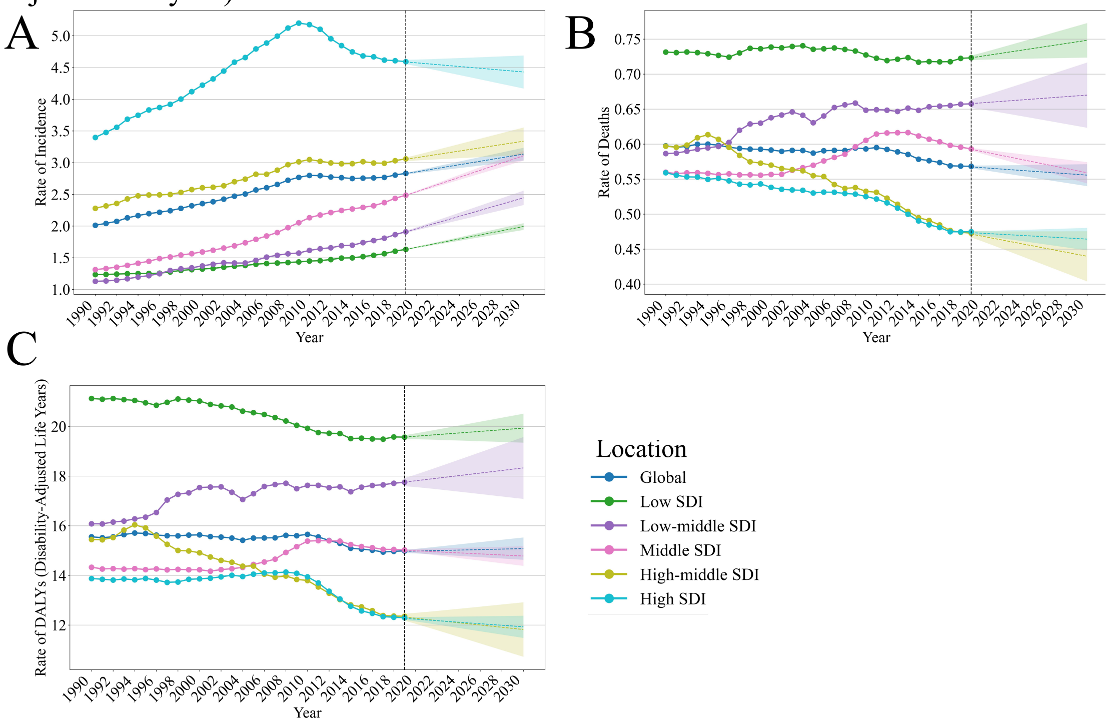

Figure S10. The correlation of SDI and global Thyroid Cancer rate from 1990 to 2019, by regions. Panel A: ASIR (age-standardized incidence rate); Panel B : ASDR (age-standardized death rate); Panel C: DALY (disability-adjusted life-year).

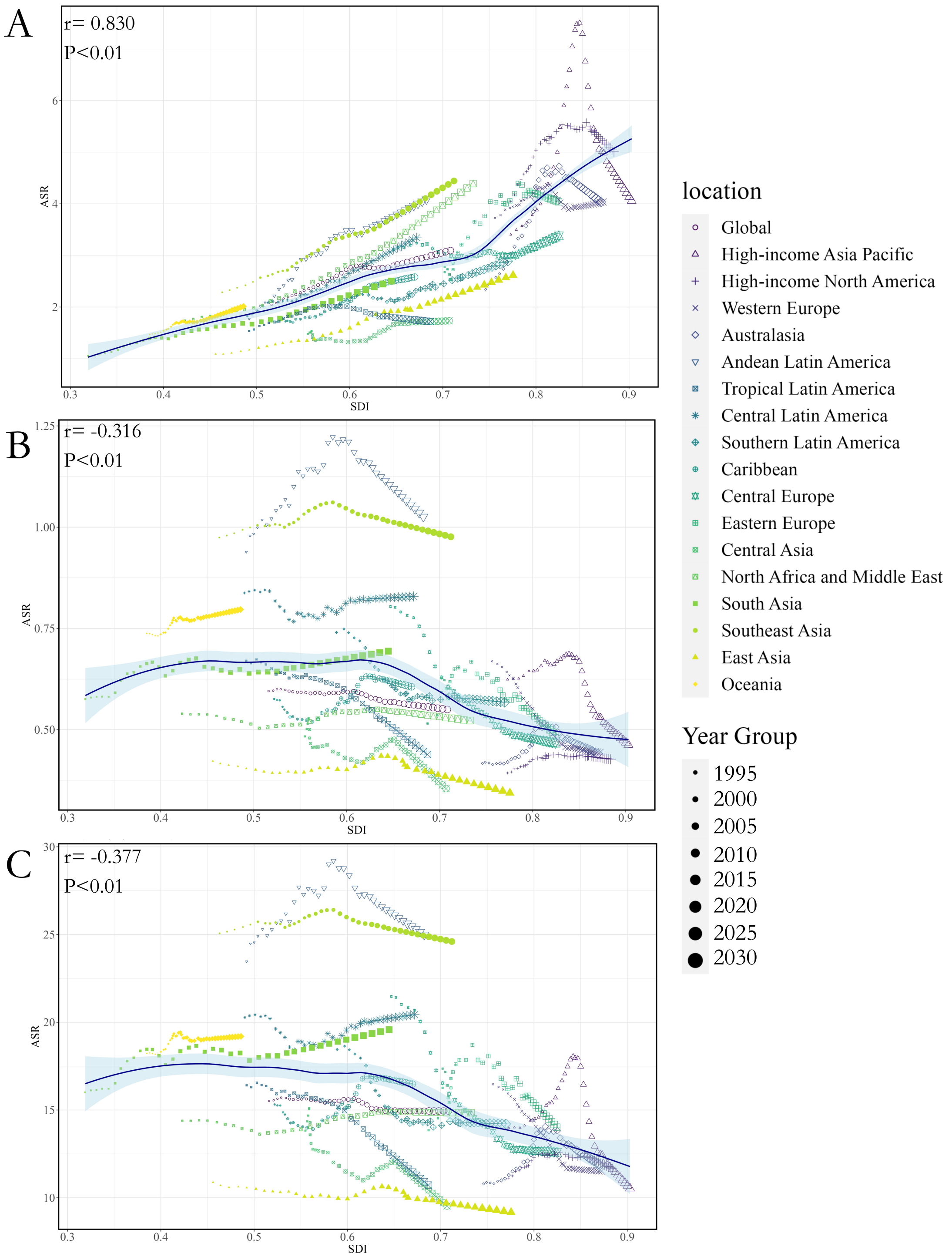

Figure S11. The correlation of SDI and EAPC in global thyroid cancer burden from 1990 to 2030, by countries. Panel A: ASIR (age-standardized incidence rate); Panel B : ASDR (age-standardized death rate); Panel C: DALY (disability-adjusted life-year).

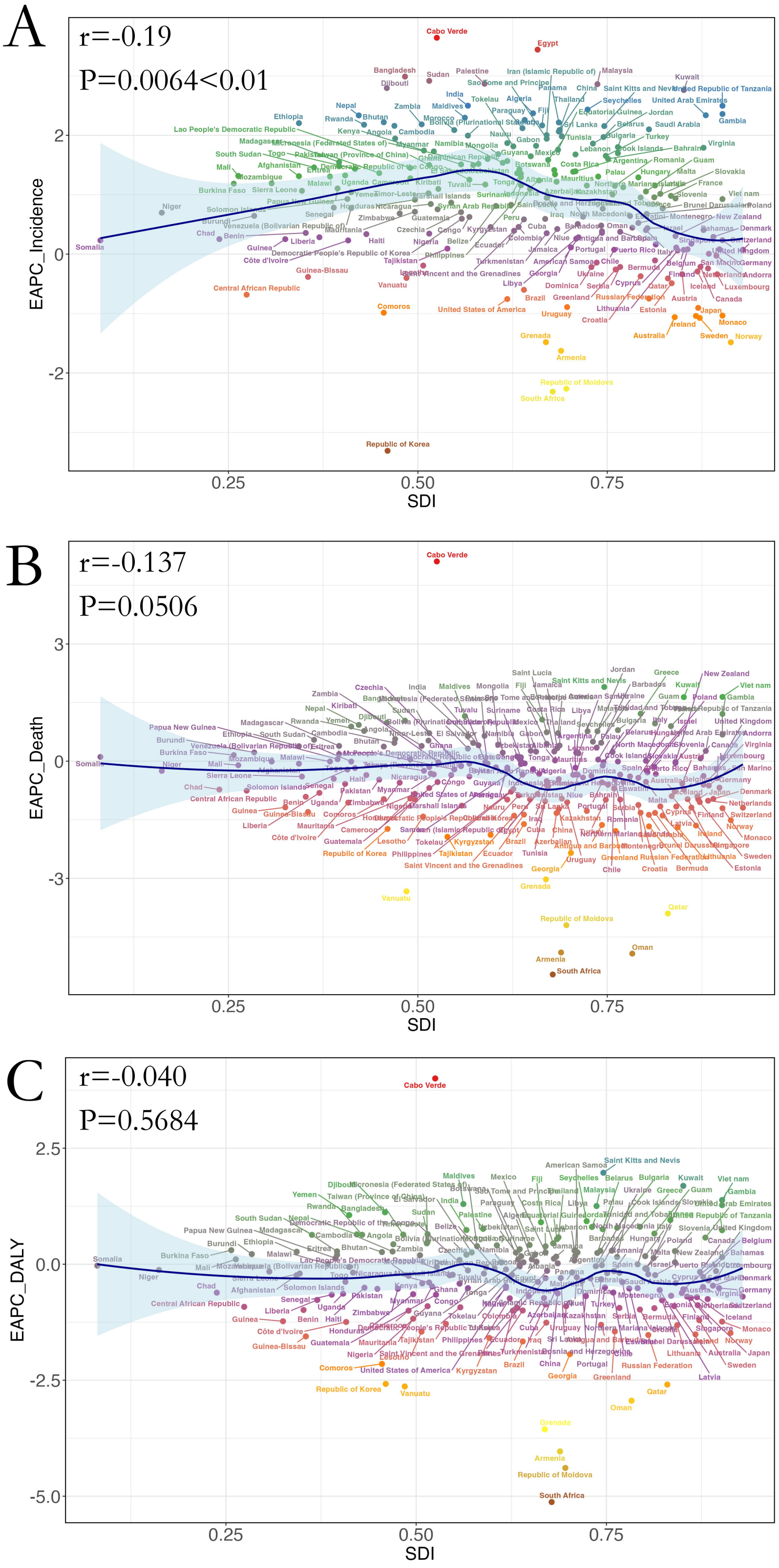

Supplement: Online Supplementary Document [file jogh-14-04090-s001.pdf]
